# Symmetry-Adapted Perturbation Theory Based on Multiconfigurational Wave Function Description of Monomers

**DOI:** 10.1021/acs.jctc.1c00344

**Published:** 2021-08-16

**Authors:** Michał Hapka, Michał Przybytek, Katarzyna Pernal

**Affiliations:** † Institute of Physics, Lodz University of Technology, ul. Wolczanska 219, 90-924 Lodz, Poland; ‡ Faculty of Chemistry, University of Warsaw, ul. L. Pasteura 1, 02-093 Warsaw, Poland

## Abstract

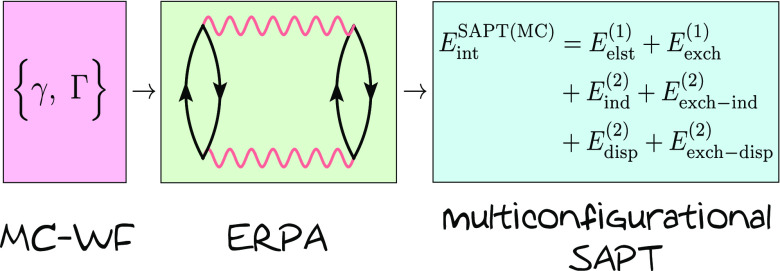

We present a formulation of the multiconfigurational (MC) wave function symmetry-adapted perturbation theory (SAPT). The method is applicable to noncovalent interactions between monomers which require a multiconfigurational description, in particular when the interacting system is strongly correlated or in an electronically excited state. SAPT(MC) is based on one- and two-particle reduced density matrices of the monomers and assumes the single-exchange approximation for the exchange energy contributions. Second-order terms are expressed through response properties from extended random phase approximation (ERPA). The dispersion components of SAPT(MC) have been introduced in our previous works [HapkaM.J. Chem. Theory Comput.2019, 15, 1016−10273052559110.1021/acs.jctc.8b01058; HapkaM.J. Chem. Theory Comput.2019, 15, 6712–67233167095010.1021/acs.jctc.9b00925]. SAPT(MC) is applied either with generalized valence bond perfect pairing (GVB) or with complete active space self-consistent field (CASSCF) treatment of the monomers. We discuss two model multireference systems: the H_2_ ··· H_2_ dimer in out-of-equilibrium geometries and interaction between the argon atom and excited state of ethylene. Using the C_2_H_4_* ··· Ar complex as an example, we examine second-order terms arising from negative transitions in the linear response function of an excited monomer. We demonstrate that the negative-transition terms must be accounted for to ensure qualitative prediction of induction and dispersion energies and develop a procedure allowing for their computation. Factors limiting the accuracy of SAPT(MC) are discussed in comparison with other second-order SAPT schemes on a data set of small single-reference dimers.

## 1 Introduction

Quantum chemistry offers two complementary approaches to noncovalent interactions, the supermolecular approach and energy decomposition methods. The former is conceptually simple and capable of providing the most accurate potential energy surfaces, e.g., for interpretation of experiments carried out in the cold- and ultracold regimes.^1−3^ The latter, decomposition methods, allow insight into the nature of the interaction by partitioning the interaction energy into well-defined contributions. The symmetry-adapted perturbation theory (SAPT)^4,5^ can be considered one of decomposition methods—it provides representation of the interaction energy as a sum of directly calculated components with a clear physical interpretation. Modern SAPT methods not only serve as interpretative tools for systems as large as enzymes exceeding 3000 atoms,^6^ but have also been applied to generate potential energy surfaces for quantitative predictions, e.g., calculations of scattering cross-sections, predictions of spectra and bulk matter properties, as well as the development of force fields for biomolecules (see, e.g., refs (7−11)).

In contrast to the rich toolbox dedicated to single-determinantal wave functions,^12,13^ describing intermolecular interactions in complexes that demand multiconfigurational (MC) wave functions presents a challenge. The multiconfigurational treatment is often mandatory for transition-metal complexes, open-shell systems, electronically excited states, or systems dominated by static correlation effects. From the standpoint of weak intermolecular forces, proper representation of static correlation, warranted by expansion in multiple electron configurations, is not sufficient. The main difficulty lies in the recovery of the remaining dynamic correlation both within and between the interacting molecules. The latter effect, giving rise to the attractive dispersion interaction, poses a particular challenge due to its highly nonlocal and long-range nature. Although many multireference methods restoring dynamic correlation effects have been developed, neither has yet managed to combine the accuracy and efficiency required for noncovalent interactions.

Application of multireference approaches in supermolecular calculations is often difficult due to the limitations of the methods themselves. For instance, the accuracy of the popular multireference configuration interaction (MRCI) approach^14^ and multireference perturbation theories^15,16^ is limited by the lack of triple excitations and truncation of the perturbation series at the second-order, respectively. Moreover, MRCI is not size-consistent and requires approximate corrections added a posteriori.^17,18^ In perturbation theories, the fulfillment of the strict separability condition depends on the choice of the zeroth-order Hamiltonian.^19,20^ A separate problem encountered in the perturbation theories, including complete active space (CAS) perturbation theory (CASPT2)^15^ and multireference variants of the Møller–Plesset perturbation theory,^21^ is the presence of intruder states,^22^ which have to be removed using one of the available shift techniques.^23^ Intruder states also present a significant difficulty in the development of multireference coupled-cluster theories,^24,25^ next to numerical instabilities and algebraic complexity. Single-reference coupled-cluster (CC) approaches introduced by Piecuch and co-workers, e.g., the CC(*P*;*Q*) formalism,^26,27^ may be a viable alternative, as indicated by studies of interactions involving stretched intramonomer covalent bonds.^28,29^ Encouraging results have recently been obtained for strongly correlated interacting systems from multiconfigurational random phase approximation theory combined with generalized valence bond method.^29−31^ The multiconfiguration density functional theory (MC DFT)^32,33^ methods corrected to include long-range dynamic correlation via perturbation theory,^34^ the adiabatic connection formalism,^35^ or semiempirical dispersion models^36^ are also worth mentioning, but their accuracy for noncovalent interactions remains to be rigorously assessed.

The SAPT formalism offers several important advantages, which make it one of the most widely used and actively developed approaches to noncovalently bound complexes.^11^ Compared to the supermolecular approach, SAPT avoids the basis set superposition error since the interaction energy is computed directly based only on monomer properties. The most accurate variants of SAPT predict interaction energies closely matching the coupled-cluster singles-and-doubles with perturbative triples [CCSD(T)^37,38^] results. Last but not least, the interaction energy represented as a sum of energy contributions is per se size-consistent.

In more than 40 years spanning the development of SAPT, applications going beyond the single-reference treatment of the monomers have been scarce. Exact full configuration interaction (FCI) wave functions are feasible only for model, few-electron dimers and have been employed in studies of SAPT convergence.^7,39−42^ Reinhardt^43^ used valence bond (VB) wave functions to represent the electrostatic interaction between monomers of a multireference character and proposed an approximate, VB-based approach for dispersion energy calculations. The spin-flip SAPT (SF-SAPT)^44,45^ formalism introduced by Patkowski and co-workers opened the possibility to treat multireference, low-spin states based on single-reference description of the subsystems. First-order spin-flip exchange energy expressions for high-spin restricted open-shell Hartree–Fock (ROHF) wave function have already been derived and implemented,^44,45^ while extension to the second-order is underway.^11^

The purpose of the present paper is to present a complete SAPT formalism applicable to interactions involving multireference systems. First steps in these directions have already been taken. Recently, we have devised multiconfigurational approaches for second-order dispersion^46^ and exchange–dispersion^47^ energy calculations. In this work, we use the same methodology to derive the second-order induction energy expressions and present formulas for the first-order electrostatic and exchange energies. The energy contributions up to the second-order in the intermolecular interaction operator constitute a variant of SAPT based on MC wave functions, which we refer to as SAPT(MC). The method can be applied with any wave function model, which gives access to one- and two-electron reduced density matrices of the monomers. Following the developments of refs (46) and (47), the linear response properties required for second-order terms are accessed by solving extended random phase approximation (ERPA)^48,49^ equations. Both first- and second-order exchange terms are derived assuming the single-exchange approximation,^50^ also known as the *S*^2^ approximation. We discuss the performance of SAPT(MC) combined with either generalized valence bond perfect pairing (GVB) or complete active space self-consistent field (CASSCF) description of the monomers.

The presented SAPT(MC) formulation is valid for the interaction between monomers in spin singlet states. The formalism may be extended to monomers with nonzero spins, which couple to the high-spin state of the dimer. For single-reference wave functions, the open-shell SAPT(MC) will become equivalent to the SAPT(ROHF) method of Żuchowski and co-workers.^51^ As all currently available many-electron SAPT approaches, SAPT(MC) is based on nondegenerate perturbation theory and is not applicable to dimers in degenerate states.

This work is organized in five sections. In Section 2, we present formulas for first- and second-order energy contributions in the ERPA-based variant of multiconfigurational SAPT. Special attention is paid to calculations of induction and dispersion energies for complexes involving electronically excited molecules. Section 3 contains details of our implementation and computations. Results for the model multireference and single-reference dimers are presented in Section 4. In Section 5, we summarize our findings.

## 2 Theory

Consider a weakly interacting dimer *AB*, which dissociates into monomer *A* in state *I* described with the |Ψ_*I*_^*A*^⟩ wave function and monomer *B* in state *J* described with the |Ψ_*J*_^*B*^⟩ wave function (*I* and *J* refer to either ground or excited states of the monomers). When the unperturbed Hamiltonian is chosen as the sum of Hamiltonians of the isolated monomers, *Ĥ*_0_ = *Ĥ*_*A*_ + *Ĥ*_*B*_, the zeroth-order wave function takes a product form |Ψ^0^⟩ = |Ψ_*I*_^*A*^Ψ_*J*_^*B*^⟩. In this work, we assume that |Ψ^0^⟩ is nondegenerate.

The intermolecular interaction operator, *V̂*, represents the perturbation and gathers all Coulombic interactions between electrons and nuclei of the interacting partners
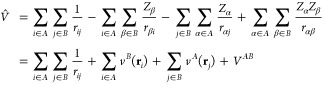
1where *i* and *j* run over *N*_*A*_ and *N*_*B*_ electrons in monomers *A* and *B*, respectively, *v*^*A*^ and *v*^*B*^ are one-electron potentials, and *V*^*AB*^ is the nuclear–nuclear repulsion term.

In the symmetrized Rayleigh–Schrödinger (SRS) formulation^52^ of SAPT, the interaction energy is expanded with respect to *V̂* while enforcing the antisymmetry of |Ψ_*I*_^*A*^⟩ and |Ψ_*J*_^*B*^⟩ wave function products. The general expression for energy contribution in the *n*th order in *V̂* takes the form
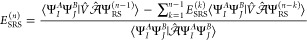
2where  is the antisymmetrizer exchanging electrons between the monomers; |Ψ_RS_^(*n*)^⟩ denotes the *n*th order component of the wave function expansion, which is identical in both SRS and the conventional Rayleigh–Schrödinger (RS) perturbation theory, i.e., the expansion is based only on simple products of zero-order functions. The difference between the SRS and RS energies is defined as the exchange energy. The RS energy contributions are often referred to as polarization components. For convenience, we use the SAPT acronym when referring to SRS.

The SAPT(MC) formalism presented in this work includes interaction energy components through the second-order in *V̂*
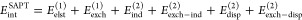
3where *E*_elst_^(1)^ and *E*_exch_^(1)^ are first-order electrostatic and exchange energy contributions, respectively, *E*_ind_^(2)^ and *E*_exch–ind_^(2)^ are the second-order induction and exchange–induction energies, respectively, and *E*_disp_^(2)^ and *E*_exch–disp_^(2)^ denote the dispersion energy and its exchange counterpart, respectively.

All formulas are in the natural orbital (NO) representation. We use the following index convention: greek μ and ν indices denote electronic states of monomers, *p*_σ_*q*_σ_*r*_σ_*s*_σ_ denotes natural spin orbitals, while *pqrs* pertains to natural orbitals denoted by φ(**r**). Throughout the work, the NOs are assumed to be real-valued. In the representation of natural orbitals, the one-electron reduced density matrix (1-RDM) is diagonal
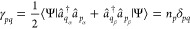
4where {*â*_*p*_σ__^†^} and {*â*_*p*_σ__} are the creation and annihilation operators, respectively, and *n*_*p*_ are the natural occupation numbers from the ⟨0,1⟩ range, summing up to half a number of electrons, ∑_*p*_*n*_*p*_ = *N*/2.

All presented SAPT(MC) energy contributions are given in a spin-summed form. The expressions for the polarization energy components are valid for arbitrary spin states of the monomers. The exchange energy contributions are presented assuming singlet spin states of the monomers, which implies that αα and ββ blocks of 1-RDM are equal.

### 2.1 First-Order Energy Contributions

The polarization component of the first-order SAPT energy is the electrostatic energy, *E*_elst_^(1)^ = ⟨Ψ_*I*_^*A*^Ψ_*J*_^*B*^|*V̂*|Ψ_*I*_^*A*^Ψ_*J*_^*B*^⟩. This energy contribution expressed in terms of 1-RDMs takes the form
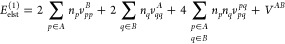
5where *v*_*pq*_^*A*(*B*)^ = ⟨φ_*p*_|*v*^*A*(*B*)^|φ_*q*_⟩ are the matrix elements of one-electron potentials and *v*_*pq*_^*rs*^ denotes the regular two-electron Coulomb integrals *v*_*pq*_^*rs*^ = ⟨φ_*p*_(**r**_1_)φ_*q*_(**r**_2_)|*r*_12_^–1^|φ_*r*_(**r**_1_)φ_*s*_(**r**_2_)⟩.

Evaluation of the exact expression for the first-order exchange energy
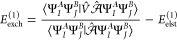
6requires access to many-particle density matrices of the monomers. For the Hartree–Fock wave function, many-particle density matrices are readily available as antisymmetrized products of the one-particle density matrix.^53^ At the SAPT(DFT) level of theory, one uses approximate 1-RDMs of the monomers based on the Kohn–Sham determinants and employs the same exchange expression as in the wave function SAPT.^54,55^

It is possible to significantly simplify the structure of eq 6 by allowing only for single exchange of electrons between the monomers in the antisymmetrizer^50,56^
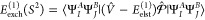
7where the single-exchange operator  collects all permutations, *P̂*_*ij*_, interchanging the coordinates of electrons *i* and *j*
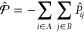
8Neglecting multiple exchange of electrons is known as the *S*^2^ approximation and allows one to express the first-order exchange energy using only 1-RDMs and two-electron reduced density matrices (2-RDMs) of the monomers.^57^ Following the density-matrix-based formulation of ref (57), we obtain
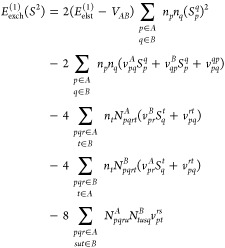
9where *S*_*p*_^*q*^ = ⟨φ_*p*_|φ_*q*_⟩ denotes the overlap integral, and we have introduced intermediates containing contractions of the 2-RDM, Γ_*p*_σ_*q*_σ′_*r*_σ″_*s*_σ‴__ = ⟨Ψ|*â*_*r*_σ″__^†^*â*_*s*_σ‴__^†^*â*_*q*_σ′__*â*_*p*_σ__|Ψ⟩, with the overlap integrals
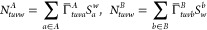
10where Γ̅_*pqrs*_ is the spin-summed 2-RDM, Γ̅_*pqrs*_ = Γ_*p*_α_*q*_α_*r*_α_*s*_α__ + Γ_*p*_β_*q*_α_*r*_β_*s*_α__. Since we assume monomers in singlet states, the ββββ + αβαβ block is equal to its αααα + βαβα counterpart.

### 2.2 Second-Order Energy Contributions

#### 2.2.1 Transition Properties from Extended Random Phase Approximation

Second-order SAPT energy components may be expressed through transition properties of the interacting monomers. The induction and dispersion energies involve transition energies and one-electron reduced transition density matrices (1-TRDMs). The SRS components, exchange–induction and exchange–dispersion energies, require both 1-TRDMs and two-electron reduced transition density matrices (2-TRDMs).

In this work, we approximate the transition properties of the interacting monomers by solving the extended random phase approximation^46,48,58^ equations (independently for each monomer)
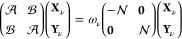
11where, in the representation of the natural spin orbitals, one obtains
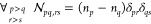
12thus, the metric matrix is diagonal and given by the occupation numbers {*n*_*p*_} (of a given monomer). For a system described with a Hamiltonian *Ĥ* and a wave function Ψ approximating a state of interest (ground or excited), the matrices  and  read
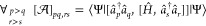
13
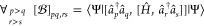
14and they are determined solely by one- and two-particle reduced density matrices of a given system. The ERPA equations may be formed as a symmetric real eigenproblem using electronic Hessian matrices,  and . For ground-state calculations, the Hessian matrices are positive definite (see, e.g., refs (59) and (60) for explicit ERPA equations in the GVB and CAS frameworks, respectively). In the case of excited-state wave functions, the Hessian matrices may have negative eigenvalues corresponding to de-excitation modes in the ERPA propagator^61^ (see a more detailed discussion in Section 2.2.3).

Apart from transition energies ω_ν_, which correspond to the poles of the ERPA eigenproblem, two quantities that are required in second-order SAPT are 1- and 2-TRDMs of the monomers. The 1-TRDM is defined as
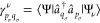
15Note that for singlet states αα and ββ blocks are equal: γ_*p*_α_*q*_α__^ν^ = γ_*p*_β_*q*_β__^ν^ = γ_*pq*_^ν^. The general definition of 2-TRDM reads
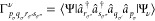
16The 1-TRDM is expressed through the ERPA eigenvectors as^60,62^
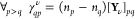
17
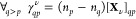
18and the formula for the half of spin-summed 2-TRDM reads^47^
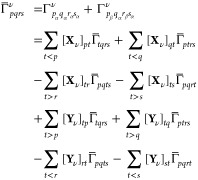
19

#### 2.2.2 Induction and Dispersion Energies

The polarization components of SAPT in the second-order are the induction and dispersion energies. The induction energy is given as
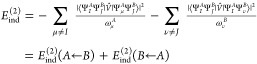
20where ω_μ_^*A*^ (ω_ν_^*B*^) are the transition energies from the state *I* (*J* for the monomer *B*) to μ (ν)
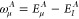
21The *E*_ind_^(2)^(*A* ← *B*) term arises from the permanent multipole moments on *B* changing the wave function of monomer *A*. The *E*_ind_^(2)^(*B* ← *A*) term describes the corresponding change in monomer *B* due to the perturbing field of *A*.

Equation 20 may be recast using contractions between 1-TRDMs of one monomer and the electrostatic potential of its unperturbed interacting partner, the latter defined as
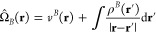
22where ρ^*B*^ is the one-electron density of the monomer *B* (analogous expression holds for Ω̂_*A*_). The total induction energy formula is now conveniently expressed as
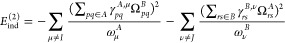
23where Ω_*pq*_ = ⟨φ_*p*_|Ω̂|φ_*q*_⟩.

In the ERPA approximation, the spin-summed formula for *E*_ind_^(2)^ takes the form
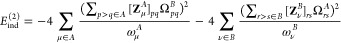
24where
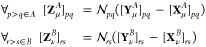
25The pertinent expression for the dispersion energy is^63,64^
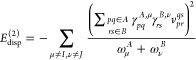
26which, in the ERPA form, reads^46^
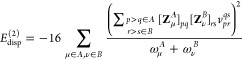
27

#### 2.2.3 Excited-State Case: Explicit Contributions to Dispersion and Induction Energies from De-Excitations

Consider a dimer *A*_*I*_*B*_0_ in an excited state, which dissociates into a monomer *A* in the state *I* > 0 denoted in this section as *A*_*I*_ (for simplicity, it is assumed that states of *A* are not degenerate) and a monomer *B* in the ground state, denoted as *B*_0_. While all transition energies, cf. eq 21, corresponding to *B*_0_ are positive
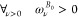
28for the monomer *A*, they take either negative or positive values for transitions to states lower or higher than *I*, respectively
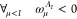
29
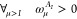
30Let us rewrite the dispersion energy expression, eq 26, in a form in which we explicitly isolate terms involving negative transitions (de-excitations)
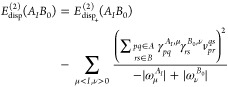
31where, by *E*_disp_+__^(2)^(*A*_*I*_*B*_0_), we denote the dispersion energy arising from the positive part of the monomer *A* linear response function spectrum
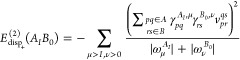
32[to emphasize that monomers A and B are, respectively, in the excited and ground states, a notation for transition properties in this subsection is changed, compared to other sections: quantities pertaining to monomers A and B are denoted as *A*_*I*_ and *B*_0_]. In eqs 31 and 32, signs of the transition energies in the denominators are written explicitly. Note that for excited states the dispersion energy may become positive-valued. This can occur either for a sufficiently high state of the monomer A (high *I*) or if the spectrum of the dimer contains states, such that |ω_μ_^*A*_*I*_^| – |ω_ν_^*B*_0_^| ≪ 1 and |ω_μ_^*A*_*I*_^| > |ω_ν_^*B*_0_^|.

Approximate methods, which are based on single-excitation operators and for which the linear response is directly related to an orbital Hessian matrix, are likely to miss de-excitations in the linear response function computed for the excited state of interest.^65^ Consequently, the second term in eq 31 would not be accounted for. Since this term involves transitions to the low-lying states, it is anticipated to give a non-negligible contribution to the dispersion energy.

A viable way to account for the de-excitations from the *A*_*I*_ state in dispersion energy calculations is by considering linear response properties of states *J* lower than *I*. After exploiting the relations connecting response properties of the states *I* and *J*
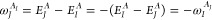
33
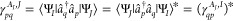
34one immediately writes the μ = *J* component of eq 31 as
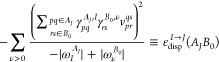
35The dispersion energy for the *A*_*I*_*B*_0_ dimer can now be written as
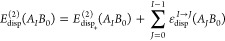
36It should be emphasized that eq 36 is fully equivalent to eq 26 if exact response properties are employed. The crucial difference between the expressions in eqs 31 and 36 is that in the former contributions to the dispersion energy from negative excitations follow from the linear response of the state *A*_*I*_, while in the latter, they are obtained from the response of states *A*_*J*_, which are lower in energy than *A*_*I*_.

The ERPA model applied to excited-state reference wave function either completely misses negative excitations or reproduces them with poor accuracy. As a result, ERPA-approximated dispersion energy, eq 27, computed for the excited-state dimer *A*_*I*_*B*_0_ will lack important contributions from de-excitations. The way around this problem is to employ the alternative formula for the dispersion energy presented in eq 36 in the ERPA approximation. This requires computing the *E*_disp_+__^(2)^(*A*_*I*_*B*_0_) term according to eq 27 and expressing the approximated ε_disp_^*I*→*J*^(*A*_*J*_*B*_0_) terms through ERPA transition properties
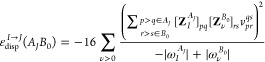
37The **Z**_*I*_^*A*_*J*_^ and ω_*I*_^*A*_*J*_^ are the *I*th eigenvector and eigenvalue, respectively, of the ERPA equations solved for the monomer *A* in the *J*th state
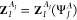
38
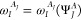
39To reiterate, the negative-energy transition *I* → *J*, which is either absent or erroneous in ERPA, is easily accessed through a positive-energy transition *J* → *I* computation carried out for the states *J* < *I*. A similar approach has recently been applied to improve the description of the correlation energy for excited states within the adiabatic connection ERPA method.^65^ Notice that for the lowest excited states, which are usually of interest, the ε_disp_^*I*→*J*^(*A*_*J*_*B*_0_) terms have a negative sign, but could, in principle, be positive for the highly excited state *I*.

The second-order induction energy for a dimer in the excited state, obtained with the ERPA approximation, eq 24, has to be corrected for the missing de-excitations in an analogous manner
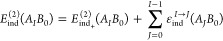
40The *E*_ind_+__^(2)^(*A*_*I*_*B*_0_) term is obtained from eq 24, where the sum with respect to μ runs through positive transitions (ω_μ_^*A*^ > 0). The ε_ind_^*I*→*J*^ (*A*_*J*_*B*_0_) terms are given as
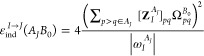
41and follow from solving ERPA equations for the monomer *A* in states lower than *I* (from the ground state, *J* = 0, up to *J* = *I* – 1). Evidently, contributions to the induction energy from negative excitations always take a positive sign.

#### 2.2.4 Second-Order Exchange Energy Contributions

We begin with the general expressions for the second-order induction and exchange–dispersion energies in the *S*^2^ approximation^66,67^
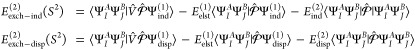
42where |Ψ_ind_^(1)^⟩ and |Ψ_disp_^(1)^⟩ are the first-order induction and dispersion wave functions, respectively
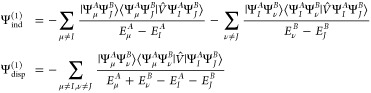
43First calculations of second-order exchange contributions in the single-exchange approximation for many-electron systems were performed by Chałasiński and Jeziorski.^67^ The authors derived general expressions in the form of a many-orbital cluster expansion based on the induction and dispersion pair functions. Expressions in terms of a one-electron orbital basis set are given in ref (68) for the exchange–dispersion energy and in ref (69) for the exchange–induction contributions. During the development of the SAPT(CCSD) approach, Korona presented the density-matrix formulation of both second-order exchange components.^70,71^

For ground-state single-determinant wave function or Kohn–Sham determinant, is it possible to calculate second-order exchange terms through all orders in the intermolecular overlap, as proven by Schäffer and Jansen.^72,73^ Recently, Waldrop and Patkowski have derived expressions for the third-order exchange–induction.^74^

The exchange–induction energy written in terms of density matrices and transition energies reads (the *S*^2^ notation is dropped for convenience)
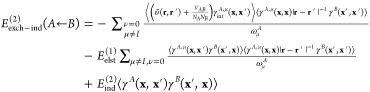
44where υ̃(**r**,**r**′) is the generalized interaction potential
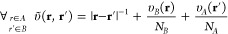
45and γ_int_^*A*,μ^ stands for the interaction density matrix^57,70^
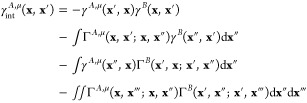
46The 1- and 2-TRDMs in the position representation are defined as
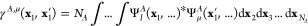
47and
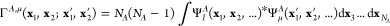
48The pertinent expressions for the *E*_exch–ind_^(2)^(*B* ← *A*) component follow by interchanging *A* and *B* indices.

In ref (47), we have derived the density-matrix formula for the exchange–dispersion energy based on transition properties in the ERPA framework. The corresponding expression for the exchange–induction energy component takes the form
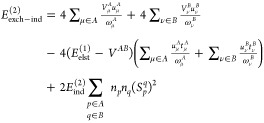
49The intermediates in eq 49 read
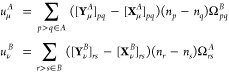
50
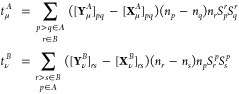
51and
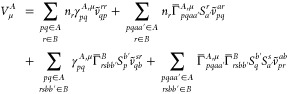
52
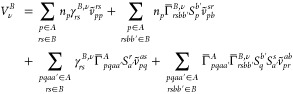
53where the effective two-electron potential (eq 45) in the matrix representation is
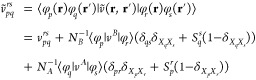
54(a φ_*p*_ orbital may belong to either monomer *X*_*p*_ = *A* or *B*).

When 1- and 2-TRDMs in eqs 52 and 53 are expanded according to eqs 17 and 18, and eq 19, respectively, one arrives at the matrix representation of the *V*_μ_^*A*^ and *V*_ν_^*B*^ terms
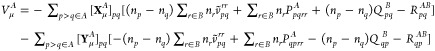
55
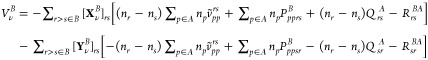
56where
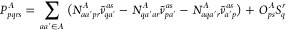
57
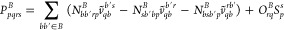
58
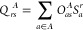
59
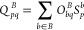
60
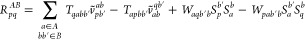
61
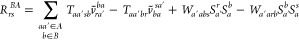
62with *N*^*A*^ and *N*^*B*^ intermediates given in eq 10 and the remaining intermediates defined as
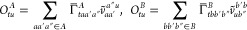
63
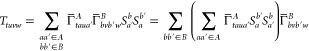
64
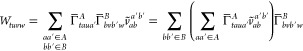
65Both induction and exchange–induction terms in SAPT are routinely calculated in the coupled approximation,^75^ so that the response of monomer orbitals due to the perturbation field of its interacting partner is accounted for. The uncoupled approach, which neglects the influence of the perturbing field, is used in calculations of the dispersion and exchange–dispersion energies in the wave function SAPT^5^ including the popular SAPT0 model.^76,77^ In both SAPT(DFT) and SAPT(CCSD), the coupled level of theory has been shown to give highly accurate second-order energy contributions.^55,70,71,78−81^

Evaluation of the exchange–induction energy requires construction of the **T** and **W** intermediates (eqs 64 and 65, respectively), which has the *n*_OCC_^6^ scaling (*n*_OCC_ are the orbitals with nonzero occupancy). Since the 2-RDM matrix elements factorize unless all four indices correspond to fractionally occupied orbitals, the formal scaling with the sixth power is only with respect to the number of such orbitals. In comparison, the exchange–dispersion energy is more expensive, as it requires steps with an *n*_OCC_^3^*n*_SEC_^3^ scaling (*n*_SEC_ are active and unoccupied orbitals).^47^ It should also be noted that the bottleneck step in evaluation of the first-order exchange energy (eq 9) engages three four-index quantities (the **N**^*A*^, **N**^*B*^ intermediates, and integrals), which amounts to scaling with the 6th power of the number of active orbitals. Note that for GVB the 2-RDMs factorize also in the active block,^48^ which results in identical scaling as in the SAPT(HF) method.

Recently, we have demonstrated that the uncoupled approximation in the ERPA framework combined with either CASSCF or GVB description of the monomers leads to a poor quality of the second-order dispersion energy.^46,47^ A more accurate dispersion energy is obtained if the monomer response properties are expanded up to the first order in the coupling parameter, which we refer to as the semicoupled approximation.^46^ The fully coupled ERPA scheme gives the best results for both dispersion and exchange–dispersion energies. In this work, all second-order energy components were obtained with the coupled approximation.

In Section 2.2.3, we demonstrated how to account for contributions from the negative transitions in second-order dispersion and induction energies calculated in the ERPA framework. Extension of this procedure to second-order exchange terms would involve first computing and storing both transition density matrices and (positive) transition energies to higher states (*J*) in the calculation for the monomer in the lower state (*I*) and then using them in a computation of the second-order exchange energies for a monomer in the higher state (*J*). The expected effect of accounting for negative transitions in the exchange–polarization energy is smaller compared to that of the polarization counterparts, and the afore-sketched procedure has not been implemented.

The multiconfigurational SAPT method, comprising first- and second-order energy components, is based on the chosen wave function theory applied to description of monomers. It is important to notice that the computation of all SAPT terms requires only knowledge of the corresponding one- and two-electron reduced density matrices of monomers. In the rest of this work, we use the notation SAPT(MC) for the proposed method, where MC indicates the underlying multiconfigurational wave function model employed to obtain reduced density matrices. The results will be presented for two multiconfigurational wave functions: CASSCF and GVB approximations. For comparison, we also include the SAPT results following from the single-determinantal description of monomers, denoted as SAPT(HF).

## 3 Computational Details

The ERPA equations applied to GVB or CAS wave functions require dividing the orbital space of each monomer into three disjoint subsets referred to as *s*_1_, *s*_2_, and *s*_3_. The cardinalities of the subsets are represented by the *M*_*s*_1__, *M*_*s*_2__, and *M*_*s*_3__ notations. For wave functions of the CAS type, the *s*_1_ set contains all inactive orbitals, whereas *s*_2_ and *s*_3_ correspond to the active and virtual orbitals, respectively. When ERPA is applied with the GVB reference, the *s*_1_ set is defined as all orbitals that occupation numbers fulfill the *n*_*p*_ > 0.992 condition. The *s*_2_ set includes all active orbitals, i.e., strongly occupied orbitals with occupation numbers 0.992 ≥ *n*_*p*_ ≥ 0.5 and their weakly occupied partners from the same geminal.^46^ The remaining orbitals are grouped in the *s*_3_ set. The *p* and *q* indices of the [**X**_ν_]_*pq*_ and [**Y**_ν_]_*pq*_ vectors span the following range
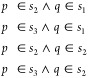
66(analogous range is assumed for the *pq* and *rs* indices of ).

In ERPA, the presence of degeneracies and near-degeneracies in the *p* ∈ *s*_2_ ∧ *q* ∈ *s*_2_ space (cf. eq 66) may lead to numerical instabilities. To circumvent this, we discarded pairs of orbitals [in practical terms, it means discarding corresponding rows and columns in the ERPA matrices (see eq 11)], applying the |*n*_*p*_ – *n*_*q*_|/*n*_*p*_ < 10^–2^ condition for the GVB wave function and |*n*_*p*_ – *n*_*q*_|/*n*_*p*_ < 10^–6^ for the CAS wave function.

The results obtained with CASSCF and GVB treatments of the monomers are denoted as SAPT(CAS) and SAPT(GVB), respectively. Pertinent calculations were performed in the locally developed code. The necessary integrals, 1- and 2-RDMs for CASSCF wave functions, were obtained from a developer version of the Molpro program.^82^ The GVB calculations were carried out in the locally modified Dalton program^83^ and interfaced with our code. The MP2 natural orbitals were used as the starting guess in both CASSCF and GVB calculations.

For the H_2_ ··· H_2_ dimer, discussed in Section 4.1, we carried out reference calculations exactly up to the second-order in SAPT, using an in-house code developed for interactions between two-electron monomers and based on the direct projection onto irreducible representations of the symmetric *S*_4_ group.^84^ The pertinent results are denoted as SAPT(FCI) in this work.

The augmented correlation-consistent orbital basis sets of double- and triple-zeta qualities (aug-cc-pV*X*Z, *X* = D,T)^85,86^ were employed throughout the work. Monomer calculations were carried out in the dimer-centered basis set.

In Section 4.3, we present results of SAPT(GVB) and SAPT(CAS) calculations for benchmark data set of noncovalently bound complexes introduced by Korona,^87^ which we refer to as the TK21 data set. The accuracy of individual SAPT energy components and interaction energies is verified against the SAPT(CCSD) benchmark. All CCSD calculations were performed with frozen core electrons. The SAPT(HF), SAPT(DFT), and SAPT2+(CCD) results are also reported. The exchange–correlation PBE0^88,89^ functional employed in SAPT(DFT) was asymptotically corrected using the GRAC scheme^90^ applied with the experimental values of the ionization potentials. The SAPT(HF), SAPT(DFT), and SAPT(CCSD) calculations were performed in Molpro.^82^ The SAPT2+(CCD) results were obtained with the Psi4^91^ program. In the latter variant of SAPT, the interaction energy is represented as
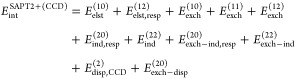
67where the (*ij*) superscript refers to the *i*th- and *j*th-order expansions in the intermolecular interaction operator and intramolecular correlation operator, respectively; the energy terms marked with the “resp” index account for the orbital relaxation effects. Except for the *E*_disp,CCD_^(2)^ term, the interaction energy components grouped in eq 67 are identical to the SAPT2^92^ approach. The “+(CCD)” notation indicates that the dispersion energy is obtained in the coupled pair approximation including noniterative contributions from single and triple excitations, here referred to as the CCD+ST(CCD)^93,94^ approach.

The accuracy of SAPT interaction energies discussed in Section 4.3 is verified against counterpoise-corrected^95^ (CP) supermolecular CCSD(T) results. To this end, we approximate higher-order induction contributions at the Hartree–Fock level of theory^96,97^
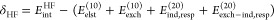
68where *E*_int_^HF^ is the supermolecular Hartree–Fock interaction energy. The δ_HF_ component was added to the SAPT interaction energy provided that the ratio of the sum of the induction and exchange–induction energies to the total interaction energy was larger than 12.5%, in agreement with the criterion selected in ref (98). Note that in Section 4.3 error statistics for total interaction energies are reported for the S_2_ subset of the TK21 data set, which excludes six largest dimers (see refs (87) and (99)).

As an additional test, we performed SAPT calculations for the A24 data set^100^ of Řezáč and Hobza. Since we observed the same qualitative trends as in the TK21 case, results for the A24 data set are given in the Supporting Information.

## 4 Results

### 4.1 Multireference Ground-State System: H_2_ ··· H_2_

We begin the analysis of multiconfigurational SAPT with a model H_2_ ··· H_2_ dimer. We monitor the change of the interaction energy upon bond dissociation in one of the hydrogen molecules. A quantitative description of this system is challenging as it has to capture the balance between long-range dynamic correlation and increasing nondynamic correlation effects.^29,101^

**Figure 1:**
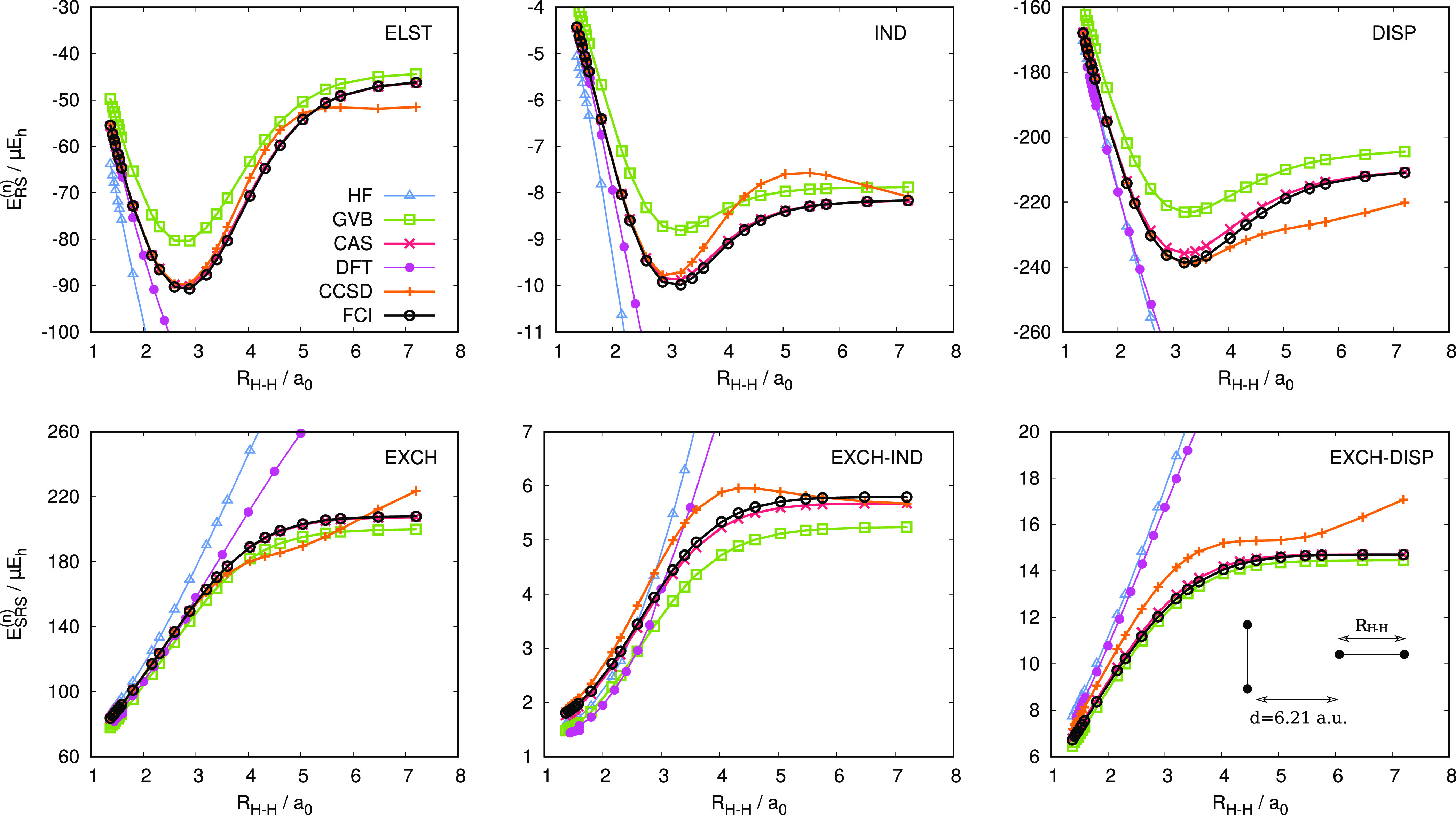
SAPT energy contributions at the Hartree–Fock (HF), GVB, CASSCF (CAS), CCSD, DFT, and FCI levels of theory for the H_2_ ··· H_2_ dimer in the T-shaped configuration. The intermolecular distance is fixed at 6.21 *a*_0_. In one of the monomers, the *R*_H–H_ distance is varied from 1.37 to 7.20 *a*_0_, while in the other monomer, the H–H bond is kept at a fixed distance of 1.44 *a*_0_. The basis set is aug-cc-pVTZ.

We examine the T-shaped structure of the H_2_ ··· H_2_ complex in which one of the covalent H–H bonds is stretched from 1.37 *a*_0_ to 7.2 *a*_0_ (see Figure 1 for a detailed description). In SAPT(CAS) calculations, each monomer is described with a CAS(2,5) wave function. Note that for two-electron monomers SAPT(GVB) is equivalent to SAPT(CAS) based on CAS(2,2) wave functions.

SAPT schemes based on either Hartree–Fock or Kohn–Sham description of the monomers fail to predict the behavior of individual interaction energy components as the H–H bond is elongated and the complex gains a multireference character (Figure 1). Although one could resort to spin-unrestricted variants of SAPT,^102,103^ breaking of the spin symmetry leads to an erroneous shape of the interaction energy components (see Figure S1 in the Supporting Information). The SAPT(CCSD) approach initially remains in excellent agreement with the SAPT(FCI) benchmark. The largest relative percent errors in SAPT(CCSD) near the equilibrium geometry (*R*_H–H_ = 1.41 *a*_0_) occur for the exchange–induction and exchange–dispersion energies, which are overestimated by ca. 4 and 7%, respectively. These discrepancies in the single-reference regime result from exclusion of certain cumulant contributions in the second-order exchange expressions.^70,71^ After the H–H bond length exceeds 3.0 *a*_0_, the XCCSD-3 approximation underlying SAPT(CCSD)^104,105^ starts to break down, which translates into qualitative errors in all interaction energy components.^29^

**Figure 2:**
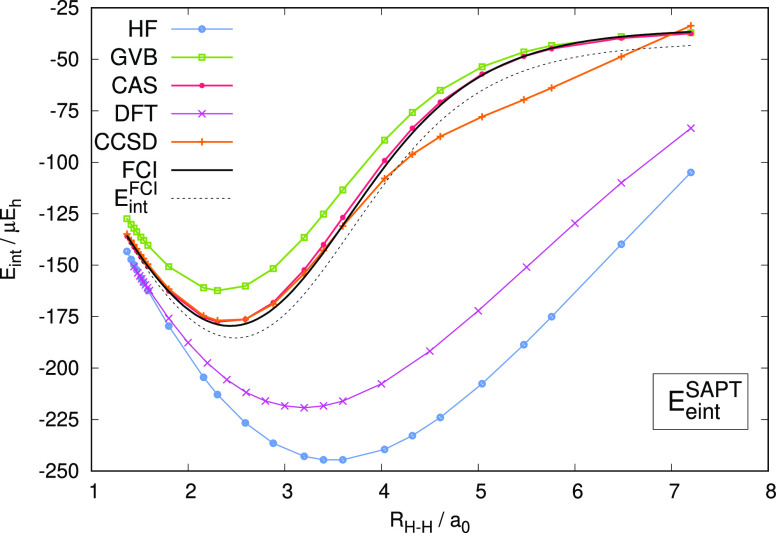
SAPT interaction energy for the H_2_ ··· H_2_ dimer in the T-shaped configuration (see Figure 1 for geometry description). *E*_int_^FCI^ denotes the supermolecular FCI interaction energy. The basis set is aug-cc-pVTZ.

Both SAPT(GVB) and SAPT(CAS) predict the correct shape of the interaction energy curves (Figures 1 and 2). The GVB-based variant systematically underestimates the magnitude of all SAPT contributions and SAPT interaction energy. The exchange–induction energy deviates most from the benchmark with relative percent errors in the 10–20% range. Errors for the remaining components stay below 12% near the equilibrium, and the accuracy improves together with the increasing share of the nondynamic correlation in the system (see also Tables S1–S3 in the Supporting Information). SAPT(CAS) is more accurate; errors with respect to the SAPT(FCI) benchmark do not exceed 3% not only in individual components, but also in the total interaction energy. The error of the SAPT(FCI) interaction energy with respect to counterpoise-corrected^95^ supermolecular FCI (denoted as *E*_int_^FCI^ in Figure 2) increases from 1.1% in the equilibrium geometry to 15% at *R*_H–H_ = 7.2 *a*_0_. The remaining discrepancy between SAPT(FCI) and supermolecular FCI reflects the role of interaction energy terms higher than second-order in the perturbation operator.

As discussed in ref (46), further extension of the active space in SAPT(CAS) is of little benefit for this system. Instead, to reach higher accuracy, one needs to move beyond the ERPA scheme and solve full linear response equations, i.e., include response not only from the orbitals but also from the wave function expansion coefficients.

### 4.2 Excited-State System: C_2_H_4_^*^ ··· Ar

**Figure 3:**
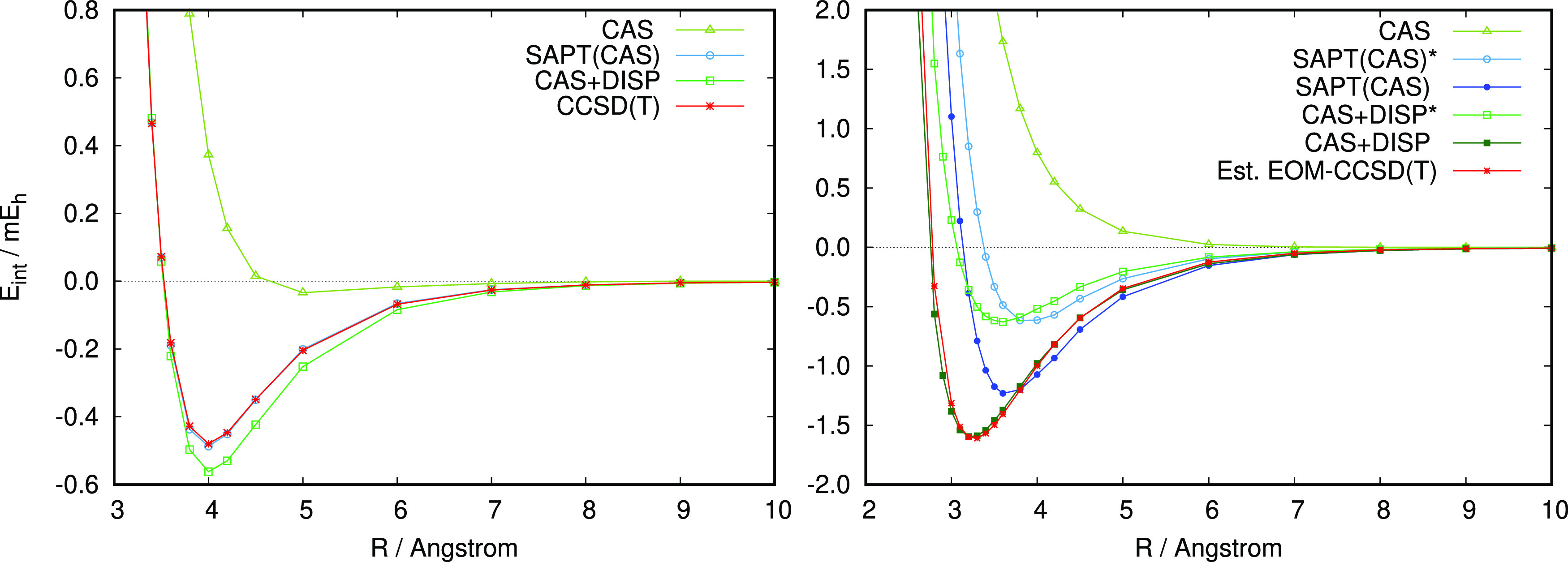
Interaction energy curves for the C_2_H_4_ ··· Ar dimer in the ground state (left) and in the π → π* state (right). Results marked with an asterisk do not include contributions from the negative energy transitions defined in eqs 37 and 41. The basis set is aug-cc-pVTZ.

In this section, we present SAPT(CAS) calculations for the C_2_H_4_ ··· Ar dimer in which the ethylene molecule is either in the ground or in the electronically excited state. We focus on the singlet excitation of a valence character with the largest contribution from the π → π* transition.^106^ The C_2_H_4_ ··· Ar complex is kept in the *C*_2*v*_ symmetry with the Ar atom located on the axis perpendicular to the C_2_H_4_ plane and bisecting its C–C bond (see also Table S4 in the Supporting Information for geometry of the C_2_H_4_ molecule). The interaction energy curves presented in Figure 3 and Table 1 are obtained by varying the distance *R* between the Ar atom and the center of the mass of ethylene. Counterpoise correction^95^ has been applied to all supermolecular interaction energies presented in this section.

**Table 1:** Components of Interaction Energy for the C_2_H_4_ ··· Ar Dimer in the Ground (g.s.) and Excited States^a^

	g.s.	π → π*	π → π*
component	*R* = 4.00 Å	*R* = 3.30 Å	*R* = 3.60 Å
*E*_elst_^(1)^	–0.257	–2.486	–1.106
*E*_exch_^(1)^	0.723	6.054	2.719
*E*_ind_+__^(2)^	–0.474	–3.987	–1.551
∑_*J*_ε_ind_^*I*→*J*^	0.000	0.000	0.000
*E*_exch–ind_^(2)^	0.457	4.434	1.813
*E*_disp_+__^(2)^	–1.026	–4.561	–2.783
∑_*J*_ε_disp_^*I*→*J*^	0.000	–1.087	–0.744
*E*_exch–disp_^(2)^	0.090	0.844	0.421
			
*E*_int_^SAPT*^	–0.487	0.298	–0.487
*E*_int_^SAPT^	–0.487	–0.789	–1.231

a *E*_int_^SAPT^ and *E*_int_^SAPT*^ are the total interaction energies with and without, respectively, the inclusion of contributions from the negative-energy transitions ε_disp/ind_^*I*→*J*^ defined in eqs 37 and 41. All values in m*E*_h_.

To access the excited-state wave functions of both the dimer and the ethylene molecule, we carried out three-state state-averaged CAS calculations (SA-CAS) in the CAS(2,3) active space, i.e., two active electrons distributed on π, σ, and π* active orbitals. In these calculations, the targeted π → π* state is the third state in the SA ensemble. Note that in both SAPT(CAS) and supermolecular CASSCF calculations the Ar atom is represented with a single determinant.

For ground-state calculations, we used supermolecular CCSD(T) results as a benchmark. To obtain reference values for excited states, we adopted the procedure of ref (107), which combines the CCSD(T) description of the ground state with excitation energies calculated at the EOM-CCSD^108^ level of theory
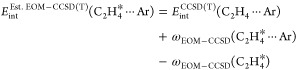
69where the asterisk indicates a molecule in the excited state, ω_EOM–CCSD_ denotes the pertinent EOM-CCSD excitation energy and *E*_int_^CCSD(T)^ is a ground-state interaction energy.

In Figure 3, we compare SAPT(CAS) interaction energy curves with supermolecular CAS(2,3) results and a coupled-cluster benchmark. As it has been rigorously shown in ref (99), supermolecular CAS interaction energy misses dispersion contributions if active orbitals are assigned only to one monomer, which is the case here. The CAS+DISP curves in Figure 3 represent CAS interaction energy supplemented with the dispersion component taken from SAPT(CAS) calculations, *E*_DISP_^(2)^ = *E*_disp_^(2)^ + *E*_exch–disp_^(2)^. For the π → π* state, both SAPT(CAS) and CAS+DISP interaction energies were computed by explicitly accounting for the de-excitation-energy terms according to eqs 36 and 40. The SAPT(CAS)* and CAS+DISP* curves were obtained by neglecting the ε_disp_^*I*→*J*^ and ε_ind_^*I*→*J*^ terms.

Inspection of Figure 3 and Table 1 shows that the C_2_H_4_ ··· Ar complex in the ground state is bound by the dispersion forces. The CAS interaction curve is mainly repulsive and features only a shallow minimum located at ca. 5.0 Å and 0.03 m*E*_h_ deep. Addition of the dispersion energy in CAS+DISP builds up a van der Waals minimum 0.56 m*E*_h_ deep localized at 4.0 Å, which is in reasonable agreement with the CCSD(T) reference (0.48 m*E*_*h*_ at *R*_eq_ = 4.0 Å). The performance of SAPT(CAS) is excellent. The total SAPT(CAS) interaction energy at the optimal monomer separation is equal to −0.49 m*E*_h_, and the entire interaction curve almost coincides with the benchmark. The dispersion energy is clearly the dominating attractive contribution amounting to −1.03 m*E*_h_ in the minimum (see Table 1).

Computation of the second-order SAPT components in the proposed SAPT(CAS) approach involves solving the ERPA equations. When the monomer reduced density matrices entering ERPA equations correspond to an unstable CAS solution, either near-instabilities or instabilities may occur in the linear response. In general, the SA-CAS calculation in a small active space bears the risk that wave functions describing higher excited states are not stable in ERPA. This is what we have encountered for the SA-CAS π → π* state of the studied C_2_H_4_ ··· Ar dimer (see Figures S2–S4). To avoid instabilities in the ERPA equations, which manifest in discontinuous interaction energy curves, we applied a three-point cubic extrapolation of second-order energy contributions based on the Dyall partitioning of the monomer Hamiltonian^109,110^ and expansion of the ERPA response properties in the coupling parameter.^46,111^ The details on the cubic extrapolation model are provided in the Supporting Information.

The interaction energy curves for the π → π* state are shown in Figure 3. At the CASSCF level of theory, the interaction has a purely repulsive character. The CAS+DISP model gives a binding curve, which remains in excellent agreement with the coupled-cluster reference. The employed CC method (eq 69) predicts a 1.61 m*E*_h_ deep minimum at an intermonomer separation of 3.3 Å. The CAS+DISP minimum occurs at a slightly shorter distance of 3.2 Å and is 1.60 m*E*_h_ deep. Note that the nearly perfect agreement with CC has to rest partially on error cancellation since CAS+DISP neglects contributions from negative excitations in the second-order exchange–dispersion energy (only ε_disp_^*I*→*J*^ terms are included in the model). The interaction energy curve from SAPT(CAS) calculations deviates from both CAS+DISP and CC results at the intermediate and short ranges. SAPT(CAS) localizes the minimum at 3.6 Å and underbinds by as much as 0.4 m*E*_h_ compared to the CC reference (Table 1). The large discrepancy between second-order SAPT and the hybrid CAS+DISP approach reflects that both higher-order induction terms and exchange contributions beyond the S^2^ approximation, present in the supermolecular CAS and absent in SAPT, become important already for the low-lying π → π* valence state.

Contributions from the negative-energy transitions in the linear response are essential for a quantitative description of the C_2_H_4_^*^ ··· Ar interaction. Neglecting the ε^*I*→*J*^ terms in SAPT reduces the well depth by a factor of 2 (cf. SAPT(CAS)* results in Figure 3). Similarly, comparing CAS+DISP with CAS+DISP* reveals that a good agreement of CAS+DISP with the coupled-cluster reference is possible only after inclusion of the de-excitation part of the spectrum. The observed energy lowering comes solely from the ε_disp_^*I*→*J*^ terms, as the induction counterparts vanish due to symmetry (Table 1). In the van der Waals minimum, the two dispersion terms (ε_disp_^2→0^ and ε_disp_^2→1^ (cf. eq 36)) sum up to −1.1 m*E*_h_, which is a sizable effect considering that positive-energy transitions amount to −4.6 m*E*_h_.

### 4.3 Single-Reference Systems

In this section, we analyze the performance of the multiconfigurational SAPT schemes for many-electron dimers of the TK21 data set of Korona^87^ against benchmark SAPT(CCSD) results. Additionally, we present SAPT(PBE0) and SAPT2+(CCD) results. Although TK21 includes systems governed by the dynamic rather than static correlation effects, our aim is to determine the level accuracy, which could be expected of the studied multiconfigurational SAPT if applied to multireference systems. Note that in all SAPT calculations the exchange terms were obtained in the *S*^2^ approximation. The first-order exchange and second-order exchange–induction contributions in SAPT(CCSD) include the cumulant contributions.^70,112^

**Figure 4:**
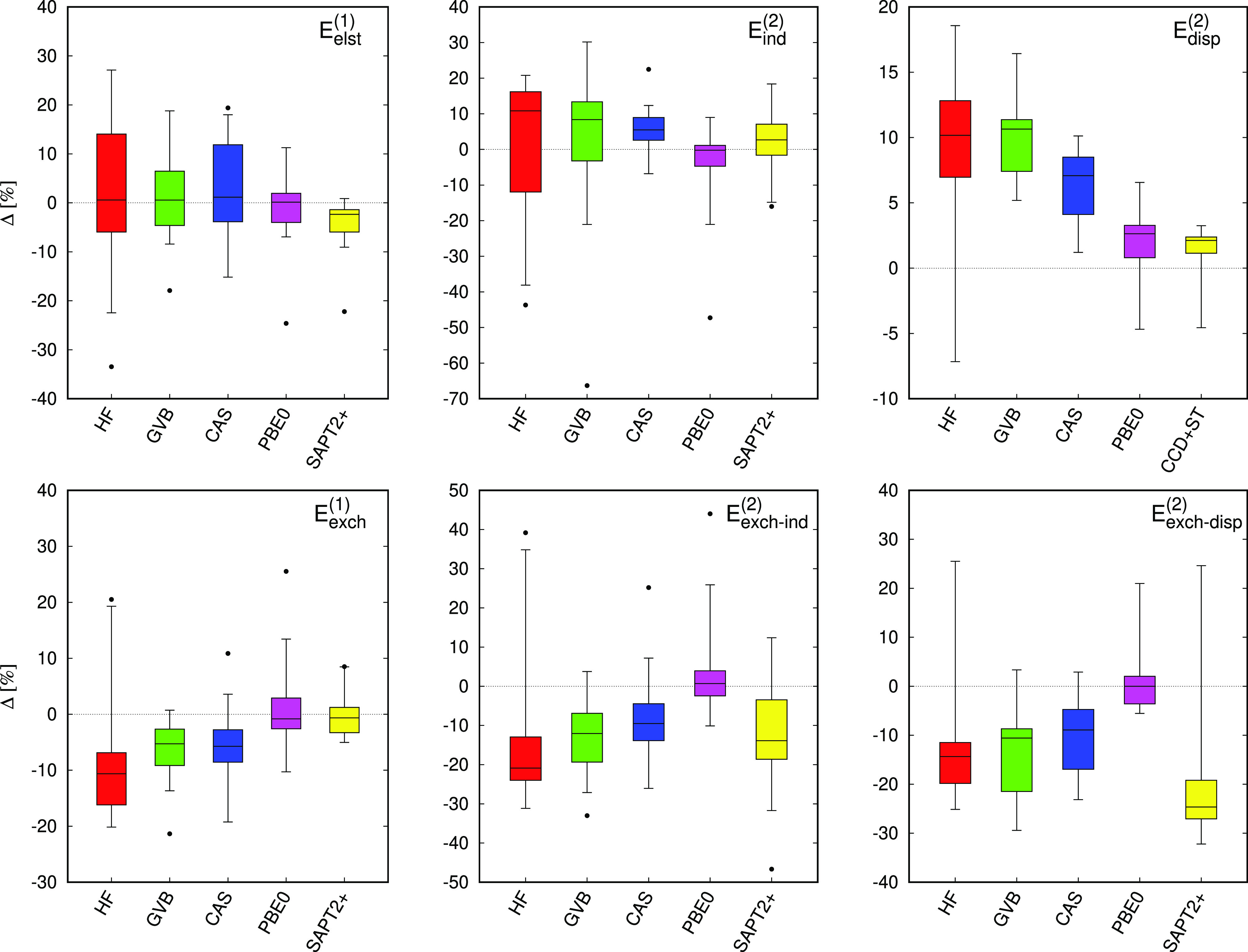
Box plots of relative percent errors (Δ) in the polarization SAPT components (top: electrostatic, induction, and dispersion energies) and in the exchange SAPT components (bottom: exchange, exchange–induction, and exchange–dispersion, all in the *S*^2^ approximation) for dimers of the TK21 data set. HF, GVB, and CAS denote wave function description of the monomers. PBE0 stands for the asymptotically corrected PBE0 functional. Relative percent errors are calculated according to the  formula. Errors are given with respect to the SAPT(CCSD) reference. Errors for the *E*_exch–disp_^(2)^ energy are reported for the S_2_ subset of TK21. The box and outer fences encompass 50 and 95% of the distribution, respectively. The outliers are marked as single points.

Figure 4 shows relative percent errors of the individual SAPT energy components with respect to the SAPT(CCSD) reference (see also Tables S8–S11 in the Supporting Information). Let us begin with the first-order energy terms. Both SAPT(GVB) and SAPT(CAS) recover the electrostatic and exchange energies with similar accuracy—the mean absolute errors (Δ̅_abs_) for these contributions fall in the 6–8% range. In contrast to the electrostatic energy, the first-order exchange is systematically underestimated with mean errors of −6.4 and −5.4% obtained with GVB and CAS wave functions, respectively. SAPT(GVB) affords a smaller spread of errors compared to SAPT(CAS), in particular, for the *E*_elst_^(1)^ component. The multireference treatment of the monomers constitutes an improvement over the Hartree–Fock (single-determinantal) description (the Δ̅_abs_ values from the Hartree–Fock-based SAPT calculations amount to ca. 13% for both components) but it remains inferior to both SAPT(DFT) and SAPT2+(CCD).

The second-order SAPT energy contributions obtained with the SAPT(CAS) variant are consistently more accurate than their SAPT(GVB) counterparts. In the TK21 data set, the largest difference occurs for the induction energy, where SAPT(GVB) deviates from the benchmark by 14.6 and 20.1% in terms of Δ̅_abs_ and standard deviation, respectively, whereas the respective errors for SAPT(CAS) amount to 7.8 and 9.5%. This confirms that the CAS-based ERPA provides a better approximation for both transition density matrices and transition energies than when GVB density matrices are used.^46,47^ Similar as in the first order, the polarization terms (*E*_ind_^(2)^ and *E*_disp_^(2)^) from multiconfigurational SAPT compare favorably with SAPT(HF), but do not match the quality of SAPT(DFT) or SAPT2+(CCD) results. The discrepancy is more pronounced for the dispersion energy where the mean absolute errors from CCD+ST(CCD) and SAPT(DFT) calculations are equal to 2.2 and 2.9%, respectively, compared to 6.4% obtained with SAPT(CAS) and 9.9% at the SAPT(GVB) level of theory.

The second-order exchange–induction and exchange–dispersion energies are more challenging than the polarization terms. SAPT(DFT) performs best, recovering the *E*_exch–ind_^(2)^ and *E*_exch–disp_^(2)^ contributions with the Δ̅_abs_ values of 7.5 and 4.3%, respectively. Both SAPT(GVB) and SAPT(CAS) tend to underestimate the second-order exchange (Δ̅_abs_ values fall in the 10–14% range). Note that SAPT2+(CCD) provides more accurate exchange–induction energy than SAPT(HF) due to the inclusion of intramonomer correlation effects via the ^*t*^*E*_exch–ind_^(22)^ term. In contrast, the exchange–dispersion energy obtained at the uncoupled level in SAPT2+(CCD) is less accurate compared to its coupled counterpart included in the SAPT(HF) approach.

In Table 2, we examine the accuracy of total SAPT interaction energies with respect to the SAPT(CCSD) reference evaluated for the S_2_ subset of the TK21 data set. Error statistics is given in terms of the mean error Δ̅, the standard deviation σ, the mean absolute error Δ̅_abs_, and the maximum absolute error Δ_max_. Both multiconfigurational SAPT approaches reach similar accuracy. With Δ̅_abs_ = 6.0% and σ = 7.4%, SAPT(GVB) remains in slightly better agreement with the benchmark than SAPT(CAS) (the respective values for the latter are Δ̅_abs_ = 6.9% and σ = 8.9%). The error statistics for multiconfigurational SAPT matches SAPT(DFT) results, where Δ̅_abs_ and σ amount to 5.6 and 7.5%, respectively. This indicates a systematic error cancellation between attractive and repulsive energy contributions in the ERPA-based SAPT since for the individual energy components, SAPT(DFT) is clearly closest to SAPT(CCSD).

**Table 2:**
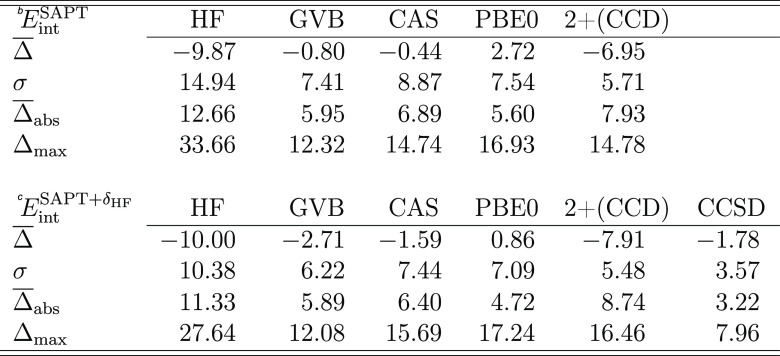
Summary of Error Statistics (in Percent) for the SAPT Interaction Energy for Dimers of the TK21/S_2_ Data Set^a^

a Errors of the SAPT interaction energy (*E*_int_^SAPT^) are given with respect to SAPT(CCSD) results. Errors of the SAPT interaction energies corrected for the δ_HF_ term (*E*_int_^SAPT+δ_HF_^) are given with respect to CP-corrected^95^ supermolecular CCSD(T) results calculated in the same basis set. The 2+(CCD) notation refers to the SAPT2+(CCD) scheme. All exchange energy components are computed in the *S*^2^ approximation. The basis set is aug-cc-pVTZ.

b Errors with respect to SAPT(CCSD).

c Errors with respect to supermolecular CCSD(T).

It is interesting to compare SAPT interaction energies against the supermolecular CCSD(T) reference. To this end, we approximate higher-order induction effects with the δ_HF_ term (eq 68). As expected, SAPT(CCSD) is the front runner (Δ̅_abs_ = 3.2%) followed by SAPT(DFT) with a mean absolute error of 4.7% (lower section of Table 2). Both SAPT(GVB) and SAPT(CAS) are less accurate—the Δ̅_abs_ value for the former reaches 5.9%, while for the latter, it amounts to 6.4%. Still, the multiconfigurational SAPT variants outperform not only the Hartree–Fock-based scheme (Δ̅_abs_ = 11.3%) but also the SAPT2 model with the CCD+ST(CCD) dispersion (Δ̅_abs_ = 8.7%). The relatively large errors of SAPT2+(CCD) can be traced to the poor representation of the second-order exchange components. Recall that the presented SAPT results neglect exchange effects beyond the *S*^2^ approximation, which is expected to worsen the agreement between SAPT and CCSD(T) interaction energies.

To summarize, the examined SAPT(GVB) and SAPT(CAS) methods benefit from a partial recovery of the intramonomer correlation effects by the underlying multiconfigurational wave function, as evidenced by a systematic improvement of all energy components with respect to the SAPT(HF) results. Nevertheless, the observed effect is small and relatively large errors compared to fully correlated SAPT schemes persist. This is best exemplified by first-order energies, which probe the quality of the monomer density (*E*_elst_^(1)^) and density matrices (*E*_exch_^(1)^). In the second-order, the accuracy of SAPT(MC) is affected by both the missing intramonomer correlation and approximations in the ERPA response equations (see also discussion in refs (46) and (47)). For the TK21 data set, we observed that both SAPT(GVB) and SAPT(CAS) tend to underestimate second-order contributions, which leads to a fortuitous error cancellation in the total interaction energy. When both TK21 and A24 data sets are considered (Table S24), SAPT(MC) predicts interaction energies with mean absolute errors and standard deviation below 8 and 10%, respectively, which is significantly better than the Hartree–Fock-based SAPT (Δ̅_abs_ ≤ 18% and σ ≤ 20%) and comparable to the SAPT2+(CCD) model (Δ̅_abs_ ≤ 10% and σ ≤ 10%).

## 5 Conclusions

We have proposed a SAPT(MC) formalism applicable to dimers in which at least one of the monomers warrants a multireference description. In the approach, the interaction energy is expanded through the second-order terms in the intermolecular interaction operator. Formulas for the exchange energy contributions are given in the single-exchange approximation (the *S*^2^ approximation) and are valid for ground and nondegenerate excited states of the monomers in spin singlet states. While singlet states require spin-free reduced density matrices, extension to high-spin dimers is straightforward and involves spin-resolved components of RDMs. Response properties that enter the density-matrix-based SAPT formulas are obtained by solving the extended random phase approximation (ERPA) eigenproblems for each subsystem. Combined with ERPA equations, the presented variant of SAPT requires access only to one- and two-electron reduced density matrices of the monomers. Note that, contrary to the supermolecular method, in SAPT the dimer wave function is never computed which is advantageous for multiconfigurational systems. In this work, we applied SAPT(MC) with either CASSCF or GVB wave functions.

Based on the model H_2_ ··· H_2_ dimer in which one of the monomers undergoes dissociation, we have verified that SAPT(MC) is capable of describing interactions in systems dominated by nondynamic correlation. The interaction energy curve from SAPT(GVB) calculations has the correct shape, and the largest deviation from the FCI benchmark does not exceed 13%. In the H_2_ ··· H_2_ dimer, several active orbitals are sufficient to recover both intra- and intermonomer correlation effects. SAPT(CAS), with only five active orbitals per monomer, predicts the total interaction energy, as well as individual energy contributions, with errors below 3% with respect to the FCI results. In contrast, SAPT schemes based on the single-reference description of the monomers, SAPT(HF) and SAPT(DFT), fail dramatically when entering the strongly correlated regime.

The proposed multiconfigurational SAPT method is the only one among the existing SAPT approaches that offers the analysis of noncovalent interactions in systems involving electronically excited molecules in singlet states. In this work, we examined the role of negative transitions in the linear response function of an excited subsystem in the description of the second-order components of SAPT. In Section 2.2.3, a general protocol for direct evaluation of negative-transition terms has been proposed and its implementation in the ERPA approximation framework has been presented. As an example of an excited-state complex, we have selected the C_2_H_4_ ··· Ar dimer and described it with a small CAS wave function. While for the ground state of the system, SAPT(CAS) remains in excellent agreement with supermolecular CCSD(T) results, the excited π → π* state of ethylene poses a significant challenge. First, we have demonstrated that second-order energy contributions related to negative excitation energies are sizable and must be accounted for in SAPT. Second, even for the low-lying valence state of ethylene, the lack of higher-order induction terms and restriction to the *S*^2^ approximation significantly limit the accuracy of SAPT(CAS) results. To illustrate this, we have presented interaction energy curves obtained in a hybrid approach, which recovers induction terms up to infinite order in *V̂*. Indeed, a combination of supermolecular CASSCF and second-order dispersion energy from SAPT(CAS) calculations, which we refer to as the CAS+DISP approach,^99^ outperforms SAPT for the π → π* state and remains in excellent agreement with the coupled-cluster reference.

The CAS+DISP hybrid can be viewed as SAPT(CAS) supplemented with a CASSCF analogue of the δ_HF_ term, i.e., the δ_CAS_ correction. These two methods become equivalent if the δ_CAS_ term is computed from a formula similar to eq 68, with CAS supermolecular energy and SAPT(CAS) energy components. While there is no advantage of SAPT(CAS) + δ_CAS_ procedure over CAS+DISP when both employ the same CAS wave functions, using CAS functions of different levels could be beneficial. Such an approach would employ CAS in the minimal active space to evaluate the δ_CAS_ term and higher-level CAS for description of monomers in SAPT(CAS). Similar to δ_HF_ correction,^113−115^ addition of δ_CAS_ would be recommended not only for excited-state complexes but also for ground-state polar systems.

To better characterize the performance of SAPT(MC) for many-electron systems, we compared different SAPT schemes against a standard single-reference data set of noncovalently bound dimers. The individual energy components from both SAPT(GVB) and SAPT(CAS) calculations are more accurate than their SAPT(HF) counterparts. This holds also for total interaction energies, where we observe a partial error cancellation between polarization and exchange terms in the second-order. The correlated SAPT schemes included in the comparison, i.e., SAPT(DFT) and SAPT2+(CCD), are systematically better than our multiconfigurational SAPT, which should be attributed to two factors. One is that ERPA-based SAPT misses the majority of dynamical correlation within the monomers as a result of employing GVB or CAS wave functions with small active spaces. Second is the quality of response properties (excitation energies and transition density matrices) from ERPA equations. Unlike the full linear response (LR-MCSCF,^116^ equivalent to MC-RPA^117^), ERPA includes response of the orbitals only.

The proposed formulation of multireference SAPT can be applied with wave function methods capable of handling large active spaces, such as density-matrix renormalization group (DMRG),^118,119^ generalized active space (GAS),^120,121^ or v2RDM-driven CAS.^122^ An efficient alternative is offered by range-separated multiconfigurational DFT.^123,124^

Without additional approximations, SAPT(MC) scales with the sixth power of molecular size. The computational bottlenecks are the solution of the full ERPA eigenproblem and evaluation of the exchange–dispersion energy formula,^47^ both involving steps with an *n*_OCC_^3^*n*_SEC_^3^ cost, where *n*_OCC_ are inactive and active and *n*_SEC_ are active and unoccupied orbitals.

A feasible path to reduce the scaling and increase the efficiency of the method with no damage to the accuracy involves density fitting or Cholesky decomposition techniques routinely applied in single-reference SAPT approaches.^76,80,92,125−128^

## Supporting Information Available

Notes on the extrapolation scheme for the second-order energy terms together with numerical illustration for C_2_H_4_^*^ ··· Ar (Figures S2–S4); SAPT(MC) interaction energy components for the H_2_ ··· H_2_ and C_2_H_4_ ··· Ar dimers (Tables S1–S3 and S5, respectively); geometry of the C_2_H_4_ molecule (Table S4); interaction energies at SAPT(CAS), CASSCF, CAS+DISP, and CC levels of theory for the Ar ··· C_2_H_4_ complex (Table S6); specification of active spaces chosen for CASSCF calculations (Table S7); individual SAPT(MC), SAPT(HF), SAPT(PBE0), SAPT2+(CCD), and SAPT(CCSD) interaction energy components together with error statistics for systems of TK21 and A24 data sets in aug-cc-pVDZ and aug-cc-pVTZ basis sets (Tables S8–S24); and Box plots of relative percent errors in the calculated SAPT energies with respect to the SAPT(CCSD) benchmark (Figures S5–S11) (PDF)
