# Supplementary material for: Symmetry-Adapted Perturbation Theory Based on Multiconfigurational Wave Function Description of Monomers
Source: J Chem Theory Comput. 2021 Aug 16;17(9):5538–55. doi: 10.1021/acs.jctc.1c00344 (PMC8444344; doi:10.1021/acs.jctc.1c00344)
Supplement: Supplementary file 1 — ct1c00344_si_001.pdf [file ct1c00344_si_001.pdf]

Supporting Information: Symmetry-adapted perturbation theory based on multiconfigurational wave function description of monomers

Michał Hapka,[*,†,‡] Michał Przybytek,[‡] and Katarzyna Pernal[†]

†*Institute of Physics, Lodz University of Technology, ul. Wolczanska 219, 90-924 Lodz, Poland*

‡*Faculty of Chemistry, University of Warsaw, ul. L. Pasteura 1, 02-093 Warsaw, Poland*

E-mail: michal.hapka@uw.edu.pl

1 Multi-reference ground-state system: $H_2 \cdots H_2$

Table S1: SAPT(GVB) interaction energies (in μHartree) for the H_2-H_2 dimer. All exchange contributions are given in the S^2 approximation. See the manuscript for geometry description. The basis set is aug-cc-pVTZ.

R_{H-H}	$E^{(1)}_{\mathrm{elst}}$	$E^{(1)}_{\mathrm{exch}}$	$E^{(2)}_{\mathrm{ind}}$	$E^{(2)}_{\mathrm{exch-ind}}$	$E^{(2)}_{\mathrm{disp}}$	$E^{(2)}_{\mathrm{exch-disp}}$	$E^{\mathrm{SAPT}}_{\mathrm{int}}$
1.3680	−49.77	77.94	−3.93	1.48	−159.64	6.46	−127.46
1.4112	−51.47	79.53	−4.09	1.50	−162.33	6.63	−130.23
1.4400	−52.59	80.61	−4.20	1.52	−164.10	6.74	−132.03
1.4688	−53.70	81.70	−4.32	1.54	−165.86	6.85	−133.79
1.5120	−55.34	83.36	−4.49	1.57	−168.47	7.01	−136.35
1.5408	−56.42	84.49	−4.60	1.59	−170.19	7.12	−138.01
1.5840	−58.00	86.19	−4.78	1.63	−172.73	7.29	−140.40
1.8000	−65.33	95.07	−5.67	1.83	−184.75	8.12	−150.72
2.1600	−74.73	110.86	−7.09	2.28	−201.82	9.49	−161.02
2.3040	−77.32	117.38	−7.58	2.50	−207.36	10.01	−162.38
2.5920	−80.30	130.46	−8.32	2.95	−215.92	10.98	−160.14
2.8800	−80.36	143.21	−8.72	3.41	−221.04	11.84	−151.67
3.2000	−77.49	156.35	−8.81	3.88	−223.11	12.62	−136.56
3.4000	−74.55	163.75	−8.74	4.14	−222.88	13.02	−125.26
3.6000	−71.11	170.38	−8.62	4.36	−221.83	13.36	−113.45
4.0320	−63.26	181.81	−8.32	4.73	−218.11	13.88	−89.28
4.3200	−58.55	187.30	−8.17	4.89	−215.41	14.10	−75.84
4.6080	−54.64	191.33	−8.06	5.01	−212.96	14.24	−65.08
5.0400	−50.36	195.27	−7.97	5.12	−210.01	14.36	−53.60
5.4720	−47.68	197.50	−7.92	5.18	−207.93	14.42	−46.43
5.7600	−46.52	198.40	−7.91	5.20	−206.94	14.44	−43.32
6.4800	−44.98	199.53	−7.89	5.23	−205.34	14.46	−38.99
7.2000	−44.39	199.92	−7.88	5.24	−204.45	14.47	−37.10

Table S2: SAPT(CAS) interaction energies (in μHartree) for the H_2-H_2 dimer. Each monomer is described with a CAS(2,5) wavefunction. See the manuscript for geometry description. The basis set is aug-cc-pVTZ.

R_{H-H}	$E^{(1)}_{\mathrm{elst}}$	$E^{(1)}_{\mathrm{exch}}$	$E^{(2)}_{\mathrm{ind}}$	$E^{(2)}_{\mathrm{exch-ind}}$	$E^{(2)}_{\mathrm{disp}}$	$E^{(2)}_{\mathrm{exch-disp}}$	$E^{\mathrm{SAPT}}_{\mathrm{int}}$
1.3680	−55.64	83.41	−4.44	1.76	−167.81	6.80	−135.93
1.4112	−57.52	85.04	−4.63	1.79	−170.71	6.96	−139.07
1.4400	−58.76	86.14	−4.75	1.81	−172.63	7.07	−141.12
1.4688	−59.98	87.26	−4.88	1.83	−174.53	7.18	−143.12
1.5120	−61.80	88.95	−5.07	1.86	−177.34	7.35	−146.04
1.5408	−62.99	90.10	−5.20	1.89	−179.19	7.46	−147.93
1.5840	−64.74	91.85	−5.40	1.93	−181.93	7.63	−150.67
1.8000	−72.86	100.91	−6.41	2.16	−194.93	8.47	−162.67
2.1600	−83.40	117.00	−8.02	2.66	−213.45	9.84	−175.37
2.3040	−86.34	123.62	−8.57	2.89	−219.47	10.37	−177.51
2.5920	−89.83	136.87	−9.40	3.38	−228.71	11.35	−176.35
2.8800	−90.06	149.71	−9.83	3.87	−234.05	12.21	−168.16
3.2000	−86.93	162.95	−9.88	4.36	−235.80	12.99	−152.30
3.4000	−83.58	170.43	−9.74	4.63	−235.10	13.38	−139.98
3.6000	−79.57	177.16	−9.53	4.86	−233.45	13.71	−126.82
4.0320	−70.17	188.85	−9.03	5.23	−228.26	14.20	−99.17
4.3200	−64.39	194.52	−8.76	5.39	−224.64	14.41	−83.47
4.6080	−59.52	198.68	−8.57	5.50	−221.41	14.54	−70.77
5.0400	−54.12	202.76	−8.38	5.60	−217.62	14.65	−57.12
5.4720	−50.66	205.05	−8.28	5.64	−215.00	14.69	−48.57
5.7600	−49.16	205.96	−8.25	5.66	−213.77	14.70	−44.86
6.4800	−47.10	207.05	−8.19	5.67	−211.84	14.71	−39.70
7.2000	−46.29	207.40	−8.17	5.68	−210.79	14.70	−37.47

Table S3: SAPT(FCI) interaction energies (in μHartree) for the H_2-H_2 dimer. See the manuscript for geometry description. The basis set is aug-cc-pVTZ.

R_{H-H}	$E^{(1)}_{\rm elst}$	$E^{(1)}_{\rm exch}$	$E^{(1)}_{\rm exch}(S^2)$	$E^{(2)}_{\rm ind}$	$E^{(2)}_{\rm exch-ind}$	$E^{(2)}_{\rm exch-ind}(S^2)$	$E^{(2)}_{\rm disp}$	$E^{(2)}_{\rm exch-disp}$	$E^{(2)}_{\rm exch-disp}(S^2)$	$E^{\rm SAPT}_{\rm int}$
1.3680	−55.49	83.64	83.61	−4.43	1.81	1.81	−167.94	6.726	6.723	−135.674
1.4112	−57.37	85.26	85.23	−4.61	1.84	1.84	−170.85	6.888	6.885	−138.839
1.4400	−58.61	86.35	86.32	−4.74	1.86	1.86	−172.77	6.997	6.993	−140.901
1.4688	−59.83	87.46	87.43	−4.87	1.88	1.88	−174.67	7.105	7.102	−142.922
1.5120	−61.65	89.15	89.12	−5.06	1.92	1.91	−177.50	7.269	7.265	−145.873
1.5408	−62.84	90.29	90.26	−5.19	1.94	1.94	−179.36	7.378	7.374	−147.785
1.5840	−64.61	92.03	91.99	−5.39	1.98	1.98	−182.12	7.542	7.538	−150.567
1.8000	−72.79	101.04	101.00	−6.41	2.22	2.21	−195.25	8.362	8.357	−162.831
2.1600	−83.51	117.04	116.98	−8.04	2.72	2.71	−214.20	9.712	9.705	−176.268
2.3040	−86.55	123.64	123.57	−8.60	2.96	2.95	−220.47	10.23	10.222	−178.796
2.5920	−90.26	136.86	136.78	−9.46	3.46	3.44	−230.30	11.195	11.185	−178.519
2.8800	−90.70	149.72	149.62	−9.92	3.96	3.94	−236.30	12.04	12.027	−171.21
3.2000	−87.71	163.03	162.91	−9.98	4.47	4.45	−238.65	12.817	12.802	−156.03
3.4000	−84.38	170.56	170.43	−9.84	4.74	4.72	−238.18	13.217	13.2	−143.872
3.6000	−80.32	177.36	177.22	−9.62	4.98	4.96	−236.59	13.552	13.534	−130.641
4.0320	−70.70	189.22	189.06	−9.09	5.36	5.34	−231.09	14.086	14.064	−102.222
4.3200	−64.75	194.98	194.81	−8.80	5.52	5.50	−227.04	14.32	14.297	−85.773
4.6080	−59.74	199.23	199.05	−8.59	5.64	5.61	−223.35	14.48	14.455	−72.333
5.0400	−54.19	203.39	203.21	−8.40	5.74	5.71	−218.93	14.62	14.594	−57.761
5.4720	−50.65	205.73	205.54	−8.29	5.79	5.76	−215.84	14.686	14.659	−48.58
5.7600	−49.12	206.65	206.46	−8.25	5.80	5.78	−214.38	14.709	14.681	−44.587
6.4800	−47.04	207.76	207.57	−8.19	5.82	5.79	−212.11	14.73	14.703	−39.03
7.2000	−46.23	208.10	207.91	−8.16	5.82	5.79	−210.91	14.733	14.705	−36.643

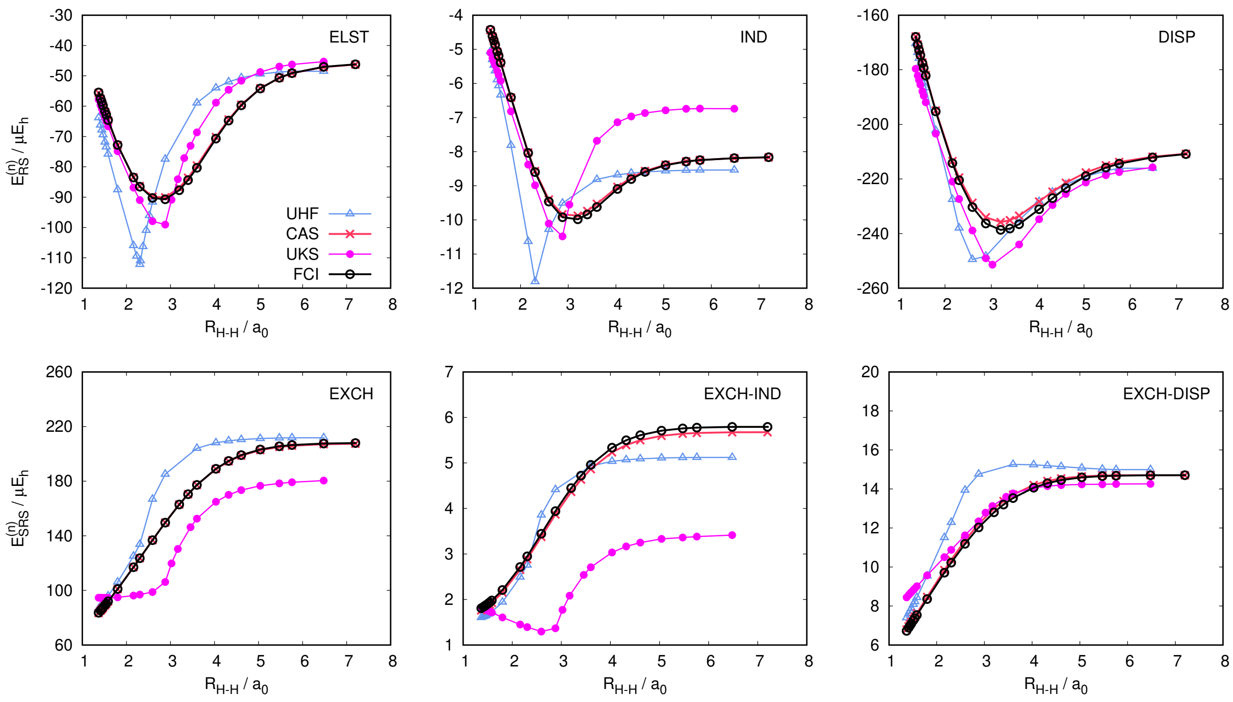

Figure S1: SAPT interaction energy for the $H_2\cdots H_2$ dimer in the T-shaped configuration. SAPT(UKS) results were performed with the asymptotically-corrected PBE0 functional.

2 Excited-state system: $C_2H_4^*\cdots Ar$

2.1 Extrapolation scheme for the second-order energy terms

In order to avoid the effect of instabilities, which may occur as a result of employing unstable SA-CAS solutions in the ERPA equations, we propose a simple three-point extrapolation scheme for computing the second-order energy contributions in SAPT(MC). The scheme is based on partitioning of the monomer X Hamiltonian (which suffers from instability) into the uncoupled component, $\hat{H}_X^{(0)}$, and the remaining part multiplied by a coupling constant α [see Eqs. (18)-(22) in Ref. Hapka et al., *J. Chem. Theory Comput.* 2019, 15, 1016]

$$\hat{H}_X = \hat{H}_X^{(0)} + \alpha \hat{H}'_X \tag{S.1}$$

The partitioned Hamiltonian is used in the ERPA equations for the monomer X. Consequently, all second-order SAPT(MC) energy terms, $E_i^{(2)}$, become functions of the coupling

parameter α. Our experience shows that potential instabilities occur for $\alpha > 0.45$, see e.g. Figs. S1-S3. The cubic extrapolation formula, which removes the instability effect, reads

$$E_i^{(2)}(\alpha) = A_i\alpha^3 + B_i\alpha + C_i \tag{S.2}$$

where

$$C_i = E_i^{(2)}(0) \tag{S.3}$$
$$B_i = \frac{E_i^{(2)}(0.01) - E_i^{(2)}(0)}{0.01} \tag{S.4}$$
$$A_i = \frac{E_i^{(2)}(\alpha_c) - E_i^{(2)}(0) - B_i\alpha_c}{\alpha_c^3} \tag{S.5}$$
$$\alpha_c = 0.45 \tag{S.6}$$

The final value of the energy component of interest $E_i^{(2)}$ used in SAPT(MC) is attained at the maximum coupling strength, $\alpha = 1$, namely $E_i^{(2)} = A_i + B_i + C_i$.

Table S4: Geometry of the C_2H_4 molecule (in angstrom).

H	0.000000	0.923274	1.238289
H	0.000000	-0.923274	1.238289
H	0.000000	0.923274	-1.238289
H	0.000000	-0.923274	-1.238289
C	0.000000	0.000000	0.668188
C	0.000000	0.000000	-0.668188

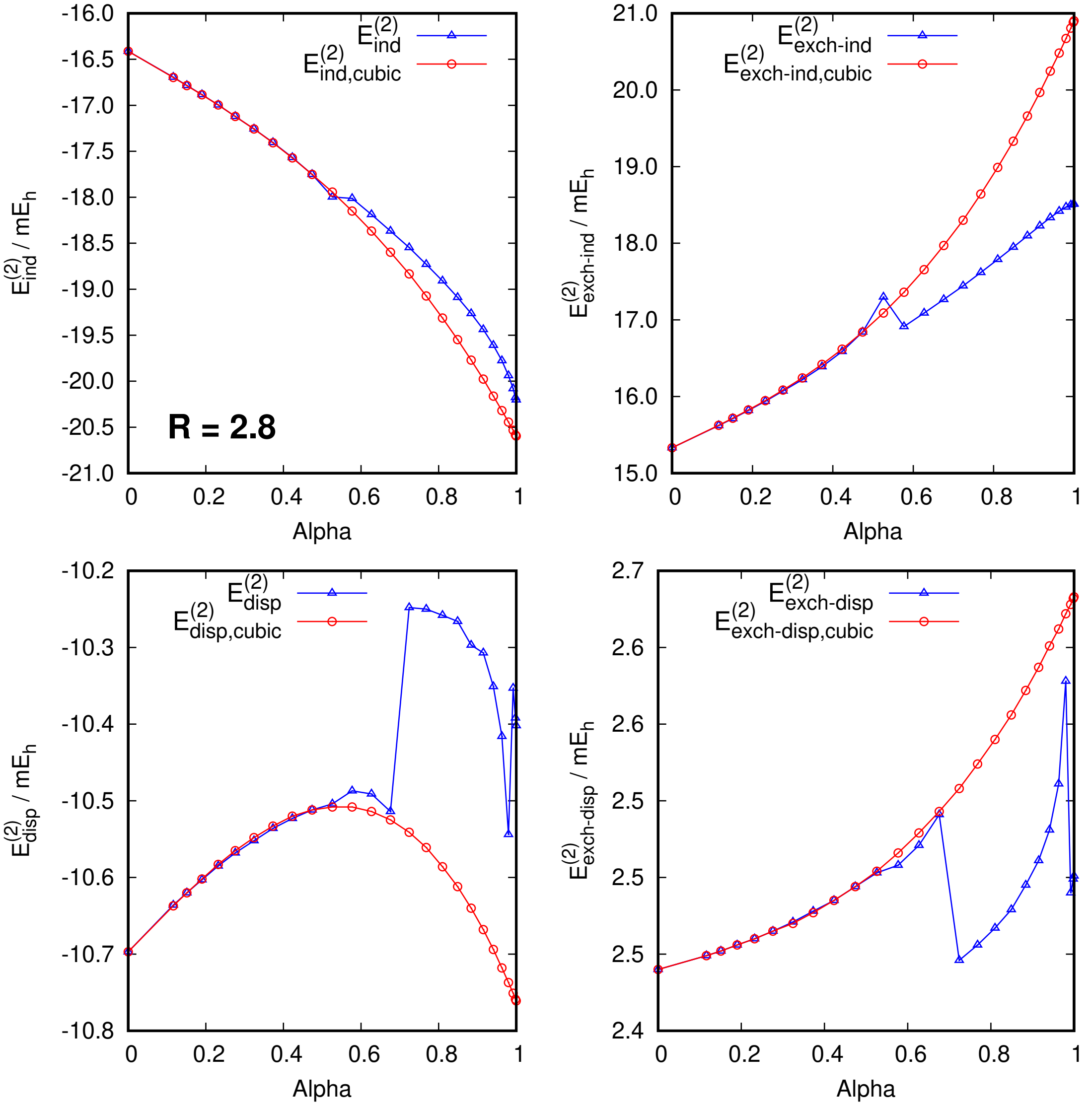

Figure S2: The α-dependence of the second-order SAPT(CAS) interaction energy components for the Ar-C_2H_4($\pi \rightarrow \pi^*$) dimer at R = 2.8 Å itermonomer separation. *Cubic* denotes extrapolation according to the $E_i^{(2)}(\alpha) = A_i\alpha^3 + B_i\alpha + C_i$ formula. The basis set is aug-cc-pVTZ.

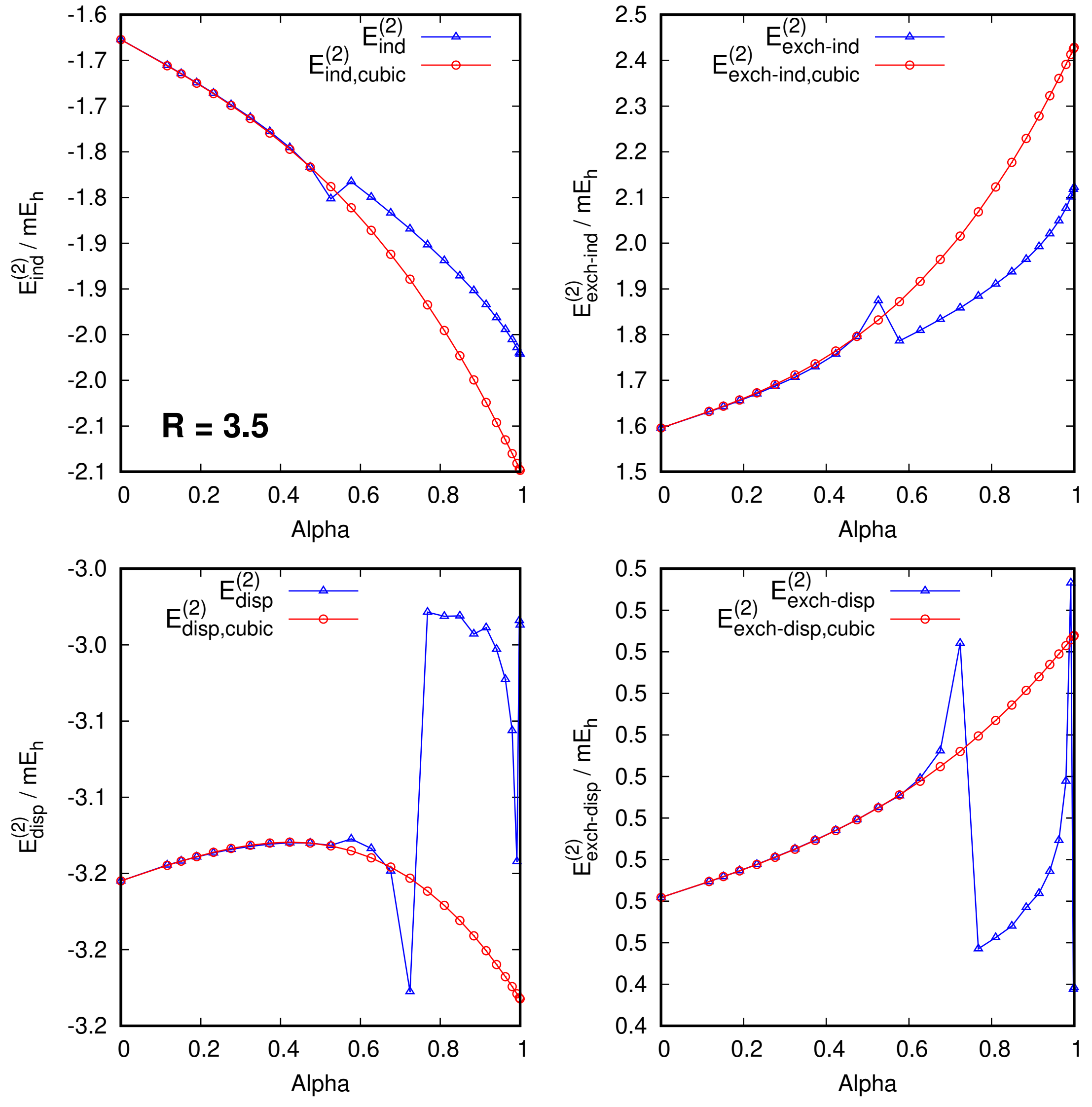

Figure S3: The α-dependence of the second-order SAPT(CAS) interaction energy components for the Ar-C_2H_4($\pi \to \pi^*$) dimer at R = 3.5 Å itermonomer separation. *Cubic* denotes extrapolation according to the $E_i^{(2)}(\alpha) = A_i\alpha^3 + B_i\alpha + C_i$ formula. The basis set is aug-cc-pVTZ.

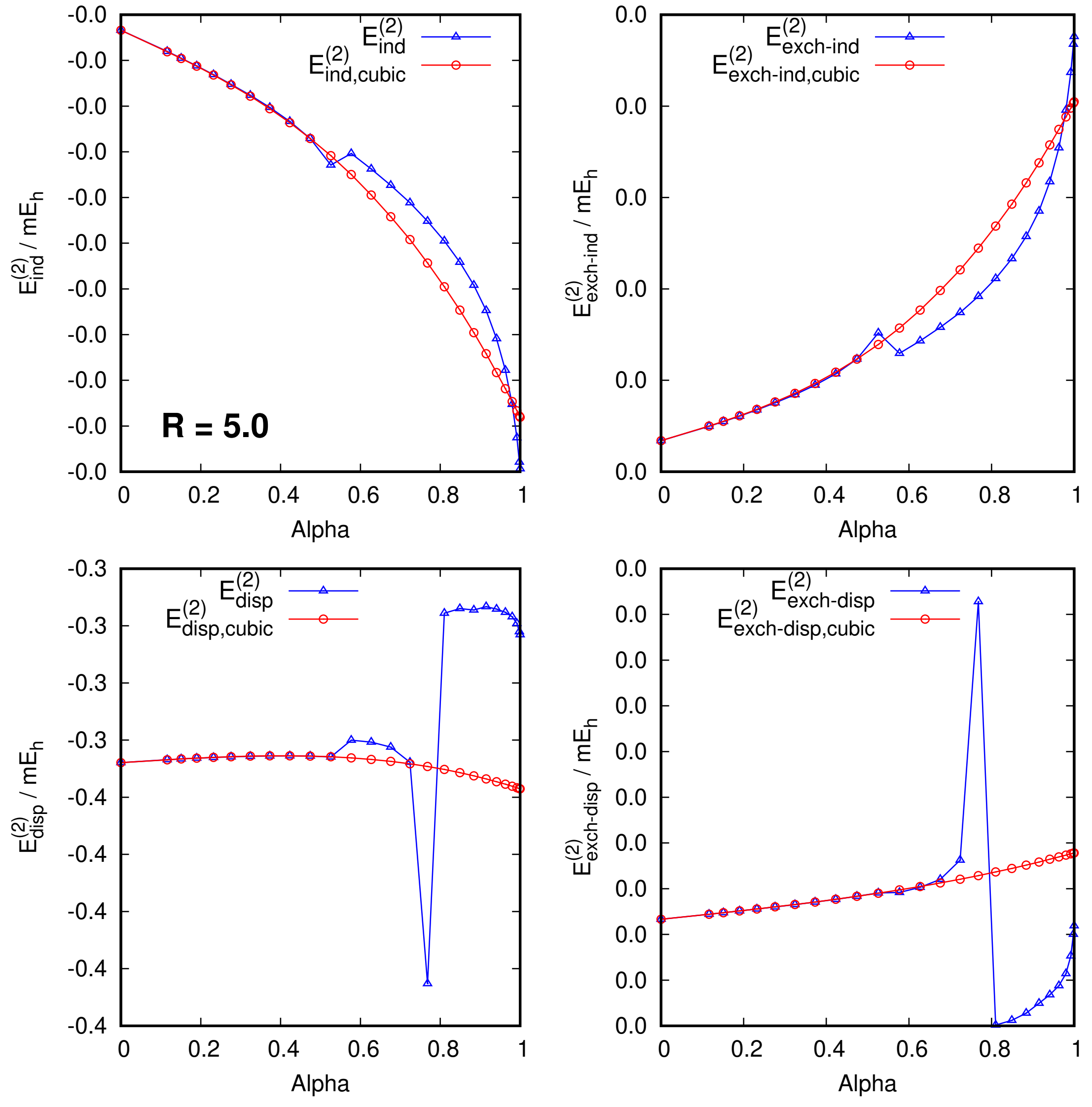

Figure S4: The α-dependence of the second-order SAPT(CAS) interaction energy components for the Ar-C_2H_4($\pi \rightarrow \pi^*$) dimer at R = 5.0 Å itermonomer separation. *Cubic* denotes extrapolation according to the $E_i^{(2)}(\alpha) = A_i\alpha^3 + B_i\alpha + C_i$ formula. The basis set is aug-cc-pVTZ.

Table S5: SAPT(CAS) interaction energy components (in milliHartree) for the Ar-$C_2H_4(\pi \rightarrow \pi^*)$ dimer. The Ar atom is described with a Hartree-Fock reference, the C_2H_4 molecule with a CAS(2,3) wavefunction. The basis set is aug-cc-pVTZ.

R	$E^{(1)}_{\rm elst}$	$E^{(1)}_{\rm exch}$	$E^{(2)}_{\rm ind}$	$E^{(2)}_{\rm exch-ind}$	$E^{(2)}_{\rm disp}$	$E^{(2)}_{\rm exch-disp}$	$\varepsilon^{20}_{\rm disp}$	$\varepsilon^{21}_{\rm disp}$	$E_{\rm int}$
2.5	−24.917	57.226	−57.830	53.925	−18.913	5.392	−2.446	−0.722	11.714
2.8	−10.325	24.416	−20.849	21.043	−10.923	2.726	−1.479	−0.632	3.976
2.9	−7.723	18.400	−14.891	15.356	−9.129	2.159	−1.244	−0.600	2.329
3.0	−5.790	13.887	−10.660	11.217	−7.647	1.708	−1.045	−0.567	1.102
3.1	−4.353	10.502	−7.654	8.209	−6.420	1.350	−0.877	−0.535	0.222
3.2	−3.284	7.962	−5.514	6.023	−5.404	1.067	−0.735	−0.503	−0.387
3.3	−2.486	6.054	−3.987	4.434	−4.561	0.844	−0.616	−0.471	−0.789
3.4	−1.889	4.619	−2.896	3.277	−3.859	0.668	−0.516	−0.440	−1.037
3.5	−1.438	3.528	−2.108	2.425	−3.267	0.529	−0.433	−0.410	−1.175
3.6	−1.106	2.719	−1.551	1.813	−2.783	0.421	−0.363	−0.381	−1.231
3.8	−0.658	1.628	−0.849	1.022	−2.026	0.266	−0.257	−0.326	−1.200
4.0	−0.399	0.992	−0.476	0.587	−1.488	0.170	−0.183	−0.277	−1.073
4.2	−0.245	0.616	−0.270	0.336	−1.115	0.110	−0.131	−0.233	−0.934
4.5	−0.122	0.311	−0.123	0.157	−0.713	0.057	−0.082	−0.179	−0.693
5.0	−0.040	0.107	−0.036	0.046	−0.361	0.020	−0.039	−0.113	−0.417
6.0	−0.005	0.015	−0.005	0.005	−0.110	0.003	−0.011	−0.045	−0.154
7.0	−0.001	0.002	−0.001	0.001	−0.040	0.000	−0.004	−0.019	−0.062
8.0	0.000	0.000	0.000	0.000	−0.017	0.000	−0.002	−0.009	−0.027
9.0	0.000	0.000	0.000	0.000	−0.008	0.000	−0.001	−0.004	−0.013
10.0	0.000	0.000	0.000	0.000	−0.004	0.000	0.000	−0.002	−0.007

Table S6: Interaction energies (in milliHartree) for the Ar-$C_2H_4(\pi \rightarrow \pi^*)$ dimer calculated at different levels of theory. In both CASSCF and SAPT calculations the Ar atom is described with a Hartree-Fock reference, and C_2H_4 with a CAS(2,3) wavefunction. SAPT* and CAS+DISP* results neglect contributions from deexcitations (see Eq. (37) and (41) in the manuscript for details). Distances given in angstrom. The basis set is aug-cc-pVTZ.

	Ar-C_2H_4(ground state)				Ar-$C_2H_4(\pi \rightarrow \pi^*)$					
R	CASSCF	CAS+DISP	SAPT	CCSD(T)	CASSCF	SAPT*	SAPT	CAS+DISP*	CAS+DISP	EOM-CCSD(T)
2.5	39.94	27.68	21.65	30.08	20.22	14.88	11.71	6.700	3.532	4.516
2.8	17.88	10.80	9.666	10.64	9.747	6.087	3.976	1.549	−0.562	−0.326
2.9					7.734	4.173	2.329	0.764	−1.080	
3.0	9.984	5.026	4.710	4.866	6.169	2.715	1.102	0.230	−1.382	−1.314
3.1	7.423	3.261	3.088	3.134	4.944	1.633	0.222	−0.127	−1.539	−1.514
3.2	5.497	1.997	1.903	1.908	3.978	0.851	−0.387	−0.359	−1.596	−1.599
3.3	4.051	1.103	1.056	1.052	3.215	0.298	−0.789	−0.502	−1.589	−1.609
3.4	2.970	0.481	0.468	0.466	2.607	−0.081	−1.037	−0.583	−1.540	−1.568
3.5	2.162	0.058	0.070	0.072	2.122	−0.333	−1.175	−0.617	−1.459	−1.497
3.6	1.562	−0.221	−0.189	−0.182	1.733	−0.487	−1.231	−0.629	−1.373	−1.406
3.8	0.789	−0.497	−0.437	−0.428	1.169	−0.617	−1.200	−0.591	−1.174	−1.203
4.0	0.373	−0.562	−0.487	−0.48	0.798	−0.614	−1.073	−0.520	−0.979	−1.000
4.2	0.156	−0.530	−0.451	−0.447	0.551	−0.569	−0.934	−0.453	−0.818	−0.818
4.5	0.015	−0.423	−0.35	−0.349	0.322	−0.433	−0.693	−0.334	−0.594	−0.595
5.0	−0.034	−0.252	−0.201	−0.204	0.136	−0.264	−0.417	−0.205	−0.358	−0.347
6.0	−0.017	−0.084	−0.066	−0.068	0.024	−0.097	−0.154	−0.083	−0.139	−0.125
7.0	−0.007	−0.032	−0.025	−0.026	0.003	−0.039	−0.062	−0.037	−0.060	−0.050
8.0	−0.002	−0.013	−0.011	−0.011	0.000	−0.017	−0.027	−0.017	−0.027	−0.022
9.0	0.000	−0.006	−0.005	−0.005	0.000	−0.008	−0.013	−0.008	−0.013	−0.011
10.0	0.000	−0.003	−0.003	−0.003	0.000	−0.004	−0.007	−0.004	−0.007	−0.006

3 Single-reference systems

Table S7: Specification of the active space selected in CAS(n, m), where n denotes the number of active electrons on m orbitals. For the GVB-PP function NGem denotes the number of geminals.

Molecule	CAS(n, m)	NGem
F^-	(10,10)	4
Na^+	(10,10)	4
H_2O	(8,8)	4
HF	(8,8)	4
CH_4	(8,8)	4
NH_3	(8,8)	4
BH_3	(6,7)	3
N_2	(10,10)	5
HCN	(6,6)	5
HCOH	(6,6)	6
C_2H_2	(4,4)	5
C_2H_4	(2,2)	6
C_2H_6	(10,10)	7
NCCN	(4,4)	9
PCCP	(4,4)	9
P_2	(6,12)	5
N_2O	(16,12)	8
CO_2	(16,12)	8
He	(2,2)	1
Ar	(8,13)	4

Table S8: Statistic of errors (in percent) of the **RS (polarization)** energy contributions obtained with various SAPT schemes for dimers of the TK21 and A24 data sets: mean error ($\overline{\Delta}$), median ($\bar{x}$), mean absolute error ($\overline{\Delta}_{\rm abs}$), standard deviation ($\sigma$) and maximum absolute error ($\Delta_{\rm max}$). MB stands for many-body SAPT2+(CCD). The PBE0 potential is corrected with the GRAC scheme. Errors are given with respect to SAPT(CCSD) results. The basis set is **aug-cc-pVDZ**.

			$E^{(1)}_{\rm elst}$					$E^{(2)}_{\rm ind}$					$E^{(2)}_{\rm disp}$		
TK21	HF	GVB	CAS	PBE0	MB	HF	GVB	CAS	PBE0	MB	HF	GVB	CAS	PBE0	MB
$\overline{\Delta}$	5.91	5.39	6.44	3.79	-5.43	5.19	7.50	8.49	1.78	3.05	7.70	10.32	6.97	3.44	0.93
$\bar{x}$	1.05	1.46	0.99	2.30	-2.91	13.20	11.09	7.91	5.46	4.88	9.08	9.64	7.41	4.49	1.81
σ	21.06	10.29	12.52	10.40	6.79	18.26	17.12	7.42	11.74	9.88	7.76	3.69	3.04	3.07	2.04
$\overline{\Delta}_{\rm abs}$	16.17	8.67	9.80	8.06	5.49	16.30	15.68	9.40	8.44	8.61	9.43	10.32	6.97	4.00	1.90
$\Delta_{\rm max}$	59.74	24.66	39.07	33.77	26.98	30.74	46.24	26.42	38.85	25.61	20.30	18.55	12.01	9.01	4.12
			$^{\dagger}E^{(1)}_{\rm elst}$					$E^{(2)}_{\rm ind}$					$E^{(2)}_{\rm disp}$		
A24	HF	GVB	CAS	PBE0	MB	HF	GVB	CAS	PBE0	MB	HF	GVB	CAS	PBE0	MB
$\overline{\Delta}$	-2.14	9.40	9.38	2.34	-5.49	0.72	15.92	12.00	2.94	-2.87	4.91	11.17	7.41	2.68	-0.59
$\bar{x}$	-2.00	9.38	7.15	0.77	-3.51	6.28	14.21	12.52	2.03	2.63	6.76	11.12	7.97	2.70	1.70
σ	14.87	11.29	11.81	8.33	5.58	18.33	8.11	8.73	2.94	10.06	7.37	2.11	2.31	2.15	2.84
$\overline{\Delta}_{\rm abs}$	12.55	11.64	11.11	5.26	5.26	16.68	15.92	12.52	3.44	8.77	7.54	11.17	7.41	2.74	2.48
$\Delta_{\rm max}$	24.98	26.12	36.23	27.42	23.88	26.95	34.98	31.69	7.62	20.65	17.09	15.01	10.67	6.20	6.37

†The dispersion-bound C_2H_4-C_2H_4 complex from the A24 dataset was excluded from $E^{(1)}_{\rm elst}$ analysis—the reference geometry is close to the point where $E^{(1)}_{\rm elst}$ crosses zero, so that relative errors become particularly large.

Table S9: Statistic of errors (in percent) of the **exchange** energy contributions obtained with various SAPT schemes for dimers of the TK21 and A24 data sets. MB stands for many-body SAPT2+(CCD). The PBE0 potential is corrected with the GRAC scheme. Errors are given with respect to SAPT(CCSD) results. **The basis set is aug-cc-pVDZ**.

	$E^{(1)}_{\mathrm{exch}}$					$E^{(2)}_{\mathrm{exch-ind}}$					$E^{(2)}_{\mathrm{exch-disp}}$				
TK21	HF	GVB	CAS	PBE0	MB	HF	GVB	CAS	PBE0	MB	HF	GVB	CAS	PBE0	MB
$\overline{\Delta}$	-11.06	-9.95	-9.40	-3.72	-0.49	-17.00	-18.33	-13.75	-4.18	-14.56	-10.62	-16.16	-12.31	-3.68	-17.77
$\bar{x}$	-15.87	-9.87	-8.01	-4.69	-1.05	-23.18	-17.65	-13.19	-8.05	-17.64	-15.70	-14.86	-11.76	-5.85	-25.00
σ	11.54	5.10	6.54	7.43	4.31	20.28	8.63	9.71	12.02	15.63	15.90	8.39	7.26	6.81	16.78
$\overline{\Delta}_{\mathrm{abs}}$	14.60	9.95	9.67	6.65	3.33	24.74	18.33	14.23	9.93	17.47	17.52	16.16	12.31	6.41	22.66
Δ_{max}	24.32	23.67	22.3	21.22	10.32	42.86	34.78	29.71	35.51	50.00	26.41	32.76	25.64	17.36	35.29
	$E^{(1)}_{\mathrm{exch}}$					$E^{(2)}_{\mathrm{exch-ind}}$					$E^{(2)}_{\mathrm{exch-disp}}$				
A24	HF	GVB	CAS	PBE0	MB	HF	GVB	CAS	PBE0	MB	HF	GVB	CAS	PBE0	MB
$\overline{\Delta}$	-6.62	-11.06	-9.72	-4.98	1.39	-9.10	-20.68	-16.56	-7.05	-6.0	-8.56	-17.44	-12.67	-3.85	-15.52
$\bar{x}$	-12.96	-9.55	-11.15	-5.28	-0.93	-23.16	-19.71	-16.67	-5.96	-12.52	-14.04	-16.47	-12.59	-3.10	-22.50
σ	11.47	5.70	5.61	2.17	5.22	21.34	8.00	9.19	3.34	14.74	13.81	5.77	5.60	3.24	13.89
$\overline{\Delta}_{\mathrm{abs}}$	11.78	11.06	10.02	4.98	4.30	21.42	20.68	16.92	7.05	14.17	14.29	17.44	12.72	3.91	18.39
Δ_{max}	21.33	23.24	20.35	8.86	11.68	32.60	37.29	34.84	16.19	29.44	26.81	28.63	22.99	11.17	30.32

Table S10: Statistic of errors (in percent) of the **RS (polarization)** energy contributions obtained with various SAPT schemes for dimers of the TK21 and A24 data sets. MB stands for many-body SAPT2+(CCD). The PBE0 potential is corrected with the GRAC scheme. Errors are given with respect to SAPT(CCSD) results, expect for $E^{(2)}_{\rm disp}$ term in A24 dataset where CCD+ST(CCD) vaules serve as reference. The basis set is **aug-cc-pVTZ**.

			$E^{(1)}_{\rm elst}$					$E^{(2)}_{\rm ind}$					$E^{(2)}_{\rm disp}$		
TK21	HF	GVB	CAS	PBE0	MB	HF	GVB	CAS	PBE0	MB	HF	GVB	CAS	PBE0	MB
$\overline{\Delta}$	1.22	1.50	3.15	-0.69	-4.17	-0.50	2.21	4.02	-4.04	1.30	9.6	9.89	6.41	2.03	1.61
$\bar{x}$	10.74	4.71	5.28	4.00	2.35	10.53	6.85	5.66	-0.23	2.69	10.17	10.73	7.08	2.63	2.14
σ	16.45	9.15	9.31	7.34	4.98	19.85	20.13	9.45	11.64	8.82	6.2	2.94	2.75	2.68	1.85
$\overline{\Delta}_{\rm abs}$	13.13	6.70	7.56	4.92	4.25	16.51	14.59	7.78	6.03	7.22	10.56	9.89	6.41	2.92	2.22
$\Delta_{\rm max}$	33.46	18.78	19.38	24.62	22.20	43.68	66.32	26.75	47.26	18.39	18.57	16.43	10.12	6.56	4.56
			$^{\dagger}E^{(1)}_{\rm elst}$					$E^{(2)}_{\rm ind}$					$E^{(2)}_{\rm disp}$		
A24	HF	GVB	CAS	PBE0	MB	HF	GVB	CAS	PBE0	MB	HF	GVB	CAS	PBE0	MB
$\overline{\Delta}$	-5.81	6.31	6.25	-1.21	-6.66	-4.50	11.67	7.65	-2.39	-3.86	2.61	8.5	4.82	0.71	
$\bar{x}$	-6.07	4.89	5.25	-1.30	-3.99	4.12	9.79	8.05	-2.54	0.59	3.49	8.80	5.58	-0.46	
σ	21.09	10.10	10.26	4.52	15.41	19.84	8.62	8.49	3.57	9.36	5.97	2.11	3.47	4.59	
$\overline{\Delta}_{\rm abs}$	14.75	9.11	9.20	2.64	9.28	17.25	11.72	9.45	3.50	7.98	5.53	8.5	5.42	2.5	
$\Delta_{\rm max}$	76.03	29.94	34.15	18.3	69.11	36.57	30.93	26.86	9.37	20.95	14.09	11.55	8.93	15.21	

†The dispersion-bound C_2H_4-C_2H_4 complex from the A24 dataset was excluded from $E^{(1)}_{\rm elst}$ analysis—the reference geometry is close to the point where $E^{(1)}_{\rm elst}$ crosses zero, so that relative errors become particularly large.

Table S11: Statistic of errors (in percent) of the **exchange** energy contributions obtained with various SAPT schemes for dimers of the TK21 and A24 data sets. MB stands for many-body SAPT2+(CCD). The PBE0 potential is corrected with the GRAC scheme. Errors are given with respect to SAPT(CCSD) results. For TK21 errors in $E^{(2)}_{\mathrm{exch-disp}}$ terms are reported only for the S_2 subset. The basis set is **aug-cc-pVTZ**.

			$E^{(1)}_{\mathrm{exch}}$					$E^{(2)}_{\mathrm{exch-ind}}$					$E^{(2)}_{\mathrm{exch-disp}}$		
TK21	HF	GVB	CAS	PBE0	MB	HF	GVB	CAS	PBE0	MB	HF	GVB	CAS	PBE0	MB
$\overline{\Delta}$	-7.15	-6.42	-5.42	1.02	-0.34	-10.35	-12.70	-8.35	2.66	-11.94	-13.50	-13.76	-9.69	0.89	-21.02
$\bar{x}$	-10.68	-5.26	-5.72	-0.83	-0.64	-17.83	-11.11	-9.51	0.71	-13.91	-13.21	-12.5	-8.7	0.0	-24.64
σ	12.12	5.39	7.16	7.47	3.79	21.66	9.92	11.37	12.59	15.39	12.02	8.78	7.62	7.05	13.45
$\overline{\Delta}_{\mathrm{abs}}$	12.91	6.50	7.16	4.70	2.83	22.45	13.35	11.75	7.52	15.85	16.90	14.21	10.19	4.31	24.30
Δ_{max}	20.51	21.36	19.57	25.56	8.50	39.16	32.77	26.05	44.54	46.66	25.52	29.41	23.16	21.05	32.20

			$E^{(1)}_{\mathrm{exch}}$					$E^{(2)}_{\mathrm{exch-ind}}$					$E^{(2)}_{\mathrm{exch-disp}}$		
A24	HF	GVB	CAS	PBE0	MB	HF	GVB	CAS	PBE0	MB	HF	GVB	CAS	PBE0	MB
$\overline{\Delta}$	-2.87	-7.49	-6.36	-0.72	1.25	-2.89	-15.6	-11.46	-0.85	-3.82					
$\bar{x}$	-9.8	-5.36	-7.29	-1.11	-0.45	-17.27	-14.19	-10.89	-1.12	-10.75					
σ	12.05	6.22	6.08	1.84	4.58	23.03	9.18	9.98	3.71	13.72					
$\overline{\Delta}_{\mathrm{abs}}$	11.43	7.72	7.74	1.73	3.73	21.94	15.6	13.29	3.11	13.12					
Δ_{max}	18.32	20.68	17.26	3.19	10.17	35.58	33.85	30.77	7.38	25.60					

Table S12: The first-order energy contributions [$E^{(1)}_{\rm elst}$ and $E^{(1)}_{\rm exch}(S^2)$, in milliHartree] for the **TK21 database**. MB stands for many-body SAPT2+(CCD). Systems from the TK21/S_2 data set are marked in gray. The basis set is aug-cc-pVDZ.

	$E^{(1)}_{\rm elst}$						$E^{(1)}_{\rm exch}(S^2)$					
	HF	GVB	CAS	PBE0	MB	CCSD	HF	GVB	CAS	PBE0	MB	CCSD
F^--HF	-121.1	-118.7	-116.1	-115.5	-114.9	-114.4	104.600	122.8	118.5	121.3	129.4	128.8
F^--H_2O	-61.22	-60.96	-61.80	-60.78	-62.11	-61.87	52.44	56.69	59.25	60.96	64.99	64.41
Na^+-H_2O	-42.55	-42.16	-41.12	-39.96	-40.11	-39.34	13.17	13.94	14.71	14.37	14.51	14.49
HF-HF	-10.12	-9.815	-9.789	-9.630	-9.782	-9.569	7.275	8.038	8.743	8.839	9.169	9.266
CH_4-CH_4	-0.235	-0.215	-0.234	-0.329	-0.296	-0.285	0.852	0.777	0.822	1.234	0.976	1.018
H_2O-H_2O	-13.60	-13.62	-13.75	-13.11	-13.60	-13.31	11.52	12.68	13.30	13.14	13.81	13.97
NH_3-CH_4	-0.848	-0.786	-0.820	-0.999	-0.971	-0.937	1.528	1.548	1.677	1.977	1.766	1.824
NH_3-H_2O	-18.46	-18.87	-18.90	-17.69	-18.48	-18.10	17.02	19.02	19.35	18.45	19.36	19.54
N_2-N_2	-0.187	-0.268	-0.278	-0.231	-0.271	-0.267	0.478	0.493	0.511	0.522	0.515	0.547
C_2H_2-C_2H_2(PD)	-2.888	-2.309	-2.471	-2.488	-2.402	-2.334	2.845	2.606	2.569	2.767	2.921	2.853
C_2H_2-C_2H_2(S)	0.246	0.192	0.214	0.206	0.126	0.154	0.773	0.621	0.587	0.628	0.748	0.678
C_2H_2-C_2H_2(T)	-3.247	-2.602	-2.782	-2.796	-2.650	-2.600	3.312	3.060	2.934	3.109	3.289	3.225
C_2H_6-HCN	-1.031	-0.864	-0.870	-1.082	-1.095	-1.026	1.739	1.739	1.747	2.091	1.958	2.067
NCCN-NCCN	-3.469	-4.025	-3.489	-3.305	-3.795	-3.524	3.599	3.691	3.516	4.014	3.947	4.101
P_2-P_2	-1.310	-1.055	-0.969	-0.967	-1.136	-1.068	3.463	2.887	2.787	2.600	3.299	3.029
N_2O-He(GM)	-0.082	-0.088	-0.082	-0.085	-0.095	-0.095	0.349	0.360	0.355	0.377	0.393	0.407
N_2O-He(LM)	-0.026	-0.031	-0.028	-0.033	-0.041	-0.036	0.148	0.171	0.152	0.183	0.188	0.192
CO_2-He(GM)	-0.068	-0.075	-0.078	-0.078	-0.086	-0.087	0.326	0.342	0.383	0.380	0.393	0.408
CO_2-He(LM)	-0.017	-0.022	-0.018	-0.024	-0.032	-0.025	0.112	0.136	0.115	0.143	0.141	0.148
Ar -Ar	-0.077	-0.081	-0.095	-0.079	-0.097	-0.093	0.259	0.274	0.312	0.267	0.305	0.308
PCCP-PCCP	-3.522	-2.943	-3.069	-2.902	-3.359	-3.144	8.222	7.421	7.229	6.948	8.098	7.747

Table S13: Induction energy components [$E^{(2)}_{\mathrm{ind}}$, $E^{(2)}_{\mathrm{exch-ind}}(S^2)$, in milliHartree] for the TK21 data set. MB stands for many-body SAPT2+(CCD). Systems from the TK21/S_2 data set are marked in gray. The basis set is aug-cc-pVDZ.

	$E^{(2)}_{\mathrm{ind}}$						$E^{(2)}_{\mathrm{exch-ind}}$					
	HF	GVB	CAS	PBE0	MB	CCSD	HF	GVB	CAS	PBE0	MB	CCSD
F^--HF	-87.88	-94.18	-99.74	-103.88	-105.99	-109.88	32.22	38.59	41.54	47.84	38.85	47.90
F^--H_2O	-35.626	-35.651	-44.084	-44.200	-44.053	-46.911	17.228	18.058	23.912	26.069	21.302	26.781
Na^+-H_2O	-22.114	-22.223	-24.804	-24.368	-24.887	-25.478	12.080	12.432	14.357	13.805	13.602	14.306
HF-HF	-3.726	-3.741	-4.310	-4.418	-4.430	-4.627	1.701	1.857	2.268	2.350	2.023	2.530
CH_4-CH_4	-0.108	-0.094	-0.102	-0.193	-0.128	-0.139	0.102	0.090	0.097	0.187	0.120	0.138
H_2O-H_2O	-4.740	-4.868	-5.692	-5.577	-5.620	-5.940	2.593	2.828	3.429	3.421	3.076	3.735
NH_3-CH_4	-0.366	-0.350	-0.396	-0.495	-0.409	-0.430	0.217	0.214	0.256	0.347	0.242	0.295
NH_3-H_2O	-7.931	-8.392	-9.607	-9.026	-8.927	-9.366	4.539	5.104	6.012	5.743	5.110	5.909
N_2-N_2	-0.067	-0.074	-0.088	-0.083	-0.079	-0.093	0.061	0.064	0.077	0.075	0.072	0.085
C_2H_2-C_2H_2(PD)	-0.877	-0.683	-0.714	-0.779	-0.834	-0.763	0.666	0.537	0.526	0.600	0.634	0.623
C_2H_2-C_2H_2(S)	-0.237	-0.172	-0.160	-0.178	-0.211	-0.183	0.172	0.125	0.113	0.128	0.153	0.141
C_2H_2-C_2H_2(T)	-1.057	-0.816	-0.918	-0.925	-0.977	-0.885	0.657	0.531	0.542	0.572	0.607	0.586
C_2H_6-HCN	-0.881	-0.732	-0.736	-0.920	-0.856	-0.841	0.264	0.242	0.237	0.358	0.256	0.334
NCCN-NCCN	-1.167	-1.267	-1.147	-1.336	-1.274	-1.425	0.765	0.830	0.753	0.982	0.835	1.068
P_2-P_2	-1.867	-1.391	-1.246	-1.304	-1.624	-1.428	1.780	1.325	1.197	1.246	1.549	1.394
N_2O-He(GM)	-0.046	-0.057	-0.045	-0.045	-0.044	-0.050	0.027	0.031	0.030	0.029	0.027	0.034
N_2O-He(LM)	-0.013	-0.019	-0.011	-0.012	-0.010	-0.013	0.007	0.008	0.008	0.009	0.005	0.010
CO_2-He(GM)	-0.050	-0.055	-0.046	-0.046	-0.046	-0.049	0.024	0.027	0.028	0.027	0.022	0.031
CO_2-He(LM)	-0.010	-0.011	-0.009	-0.010	-0.009	-0.010	0.004	0.006	0.005	0.006	0.004	0.007
Ar -Ar	-0.081	-0.086	-0.107	-0.085	-0.097	-0.104	0.080	0.084	0.105	0.084	0.095	0.102
PCCP-PCCP	-4.287	-3.527	-3.298	-3.459	-4.020	-3.720	3.959	3.328	3.053	3.225	3.712	3.517

Table S14: Dispersion energy components [$E^{(2)}_{\rm disp}$, $E^{(2)}_{\rm exch-disp}(S^2)$, in milliHartree] for the TK21 data set. MB stands for many-body SAPT2+(CCD). Systems from the TK21/S_2 data set are marked in gray. The basis set is aug-cc-pVDZ.

	$E^{(2)}_{\rm disp}$						$E^{(2)}_{\rm exch-disp}$					
	HF	GVB	CAS	PBE0	MB	CCSD	HF	GVB	CAS	PBE0	MB	CCSD
F^--HF	-17.160	-18.557	-18.947	-19.592	-21.355	-21.532	5.551	5.072	5.609	6.973	5.574	7.543
F^--H_2O	-10.661	-10.801	-12.001	-12.332	-12.970	-13.300	3.612	3.472	3.916	4.557	3.509	4.887
Na^+-H_2O	-0.395	-0.404	-0.427	-0.443	-0.438	-0.436	0.090	0.092	0.101	0.105	0.085	0.101
HF-HF	-2.249	-2.300	-2.512	-2.554	-2.664	-2.713	0.335	0.337	0.391	0.421	0.322	0.452
CH_4-CH_4	-1.418	-1.302	-1.379	-1.584	-1.485	-1.525	0.102	0.089	0.095	0.142	0.085	0.121
H_2O-H_2O	-3.675	-3.728	-4.046	-4.047	-4.217	-4.307	0.709	0.703	0.801	0.824	0.669	0.893
NH_3-CH_4	-1.620	-1.545	-1.671	-1.792	-1.737	-1.786	0.160	0.150	0.171	0.204	0.138	0.190
NH_3-H_2O	-5.016	-5.152	-5.521	-5.433	-5.599	-5.708	1.133	1.152	1.284	1.265	1.056	1.353
N_2-N_2	-0.652	-0.637	-0.651	-0.664	-0.675	-0.693	0.041	0.040	0.044	0.043	0.037	0.046
C_2H_2-C_2H_2(PD)	-2.201	-1.994	-2.167	-2.169	-2.192	-2.190	0.276	0.234	0.250	0.264	0.255	0.270
C_2H_2-C_2H_2(S)	-1.129	-0.983	-0.965	-1.040	-1.066	-1.046	0.128	0.096	0.090	0.103	0.112	0.103
C_2H_2-C_2H_2(T)	-2.169	-1.967	-2.111	-2.127	-2.154	-2.154	0.276	0.234	0.234	0.255	0.247	0.268
C_2H_6-HCN	-1.643	-1.566	-1.626	-1.777	-1.746	-1.807	0.120	0.112	0.119	0.146	0.105	0.151
NCCN-NCCN	-2.983	-2.860	-3.000	-3.071	-3.141	-3.178	0.325	0.316	0.314	0.364	0.317	0.381
P_2-P_2	-3.154	-2.796	-2.724	-2.835	-3.079	-2.971	0.494	0.382	0.350	0.370	0.471	0.393
N_2O-He(GM)	-0.429	-0.425	-0.431	-0.447	-0.458	-0.468	0.017	0.018	0.018	0.018	0.015	0.020
N_2O-He(LM)	-0.225	-0.230	-0.226	-0.238	-0.249	-0.252	0.007	0.008	0.007	0.008	0.006	0.009
CO_2-He(GM)	-0.366	-0.369	-0.382	-0.397	-0.407	-0.417	0.013	0.014	0.015	0.015	0.011	0.017
CO_2-He(LM)	-0.175	-0.178	-0.176	-0.188	-0.193	-0.197	0.005	0.005	0.005	0.006	0.004	0.006
Ar -Ar	-0.404	-0.403	-0.414	-0.414	-0.431	-0.433	0.018	0.019	0.021	0.019	0.018	0.021
PCCP-PCCP	-8.161	-7.232	-7.341	-7.551	-8.254	-7.927	1.302	1.076	1.053	1.100	1.362	1.110

Table S15: The first-order energy contributions [$E^{(1)}_{\mathrm{elst}}$ and $E^{(1)}_{\mathrm{exch}}(S^2)$, in milliHartree] for the **A24 database**. MB stands for many-body SAPT2+(CCD). The basis set is aug-cc-pVDZ.

	$E^{(1)}_{\mathrm{elst}}$						$E^{(1)}_{\mathrm{exch}}(S^2)$					
	HF	GVB	CAS	PBE0	MB	CCSD	HF	GVB	CAS	PBE0	MB	CCSD
H_2O-NH_3	-18.05	-18.45	-18.48	-17.291	-18.07	-17.70	16.34	18.26	18.58	17.55	18.58	18.75
H_2O-H_2O	-13.03	-13.02	-13.10	-12.489	-12.92	-12.64	10.54	11.60	12.13	12.00	12.61	12.73
HCN-HCN	-10.09	-9.93	-9.481	-9.248	-9.567	-9.147	6.044	6.887	6.390	6.783	6.786	7.225
HF-HF	-10.70	-10.41	-10.43	-10.18	-10.34	-10.11	7.977	8.865	9.328	9.616	10.04	10.14
NH_3-NH_3	-7.589	-7.658	-7.817	-7.426	-7.829	-7.594	6.519	6.954	7.469	7.136	7.447	7.593
HF-CH_4	-1.781	-1.457	-1.399	-2.163	-2.033	-1.967	3.464	3.524	3.635	4.051	4.159	4.251
NH_3-CH_4	-1.491	-1.456	-1.490	-1.552	-1.619	-1.564	2.101	2.420	2.592	2.281	2.413	2.503
H_2O-CH_4	-1.111	-1.016	-1.041	-1.148	-1.162	-1.121	1.534	1.631	1.779	1.712	1.778	1.802
HCOH-HCOH	-12.66	-10.85	-10.27	-10.51	-10.64	-10.24	12.24	12.80	12.42	13.16	14.04	14.08
H_2O-C_2H_4	-6.326	-5.138	-5.290	-5.424	-5.586	-5.330	7.018	6.316	6.308	6.509	7.342	6.899
HCOH-C_2H_4	-3.346	-2.609	-2.559	-2.755	-2.849	-2.703	4.069	3.730	3.693	4.005	4.311	4.204
C_2H_2-C_2H_2(1)	-3.376	-2.703	-2.746	-2.910	-2.752	-2.701	3.498	3.234	3.091	3.317	3.470	3.403
NH_3-C_2H_4	-3.090	-2.492	-2.551	-2.648	-2.766	-2.613	3.853	3.404	3.422	3.524	3.977	3.725
C_2H_4-C_2H_4(1)	-1.563	-1.281	-1.309	-1.402	-1.539	-1.440	3.460	3.038	2.988	3.237	3.575	3.400
CH_4-C_2H_4	-0.777	-0.585	-0.592	-0.686	-0.723	-0.672	1.563	1.332	1.357	1.369	1.588	1.474
BH_3-CH_4	-2.228	-1.983	-2.004	-2.442	-2.429	-2.396	5.839	5.518	5.759	6.523	6.386	6.563
CH_4-C_2H_6(1)	-0.547	-0.495	-0.529	-0.619	-0.656	-0.639	1.811	1.698	1.795	2.093	2.048	2.115
CH_4-C_2H_6(2)	-0.351	-0.319	-0.338	-0.411	-0.445	-0.432	1.270	1.169	1.213	1.505	1.464	1.523
CH_4-CH_4	-0.316	-0.288	-0.311	-0.358	-0.392	-0.379	1.134	1.038	1.094	1.314	1.292	1.344
Ar-CH_4	-0.264	-0.253	-0.268	-0.295	-0.324	-0.319	0.864	0.837	0.879	0.955	0.991	1.023
Ar-C_2H_4	-0.464	-0.368	-0.358	-0.378	-0.445	-0.407	1.237	1.008	1.000	1.037	1.240	1.129
C_2H_4-C_2H_2	-0.586	-0.573	-0.483	-0.550	-0.938	-0.758	6.386	5.203	5.068	5.400	6.223	5.688
C_2H_4-C_2H_4(2)	-0.999	-0.882	-0.780	-0.839	-1.303	-1.086	7.638	6.047	5.941	6.302	7.540	6.752
C_2H_2-C_2H_2(2)	0.513	0.299	0.388	0.329	-0.075	0.094	5.344	4.500	4.339	4.652	5.135	4.806

Table S16: Induction energy components [$E^{(2)}_{\text{ind}}$, $E^{(2)}_{\text{exch-ind}}(S^2)$, in milliHartree] for the A24 data set. MB stands for many-body SAPT2+(CCD). The basis set is aug-cc-pVDZ.

	$E^{(2)}_{\text{ind}}$						$E^{(2)}_{\text{exch-ind}}$					
	HF	GVB	CAS	PBE0	MB	CCSD	HF	GVB	CAS	PBE0	MB	CCSD
H_2O-NH_3	-7.647	-8.093	-9.270	-8.441	-8.604	-9.0273	4.371	4.920	5.800	5.245	4.918	5.6909
H_2O-H_2O	-4.329	-4.435	-5.152	-5.048	-5.099	-5.3615	2.301	2.510	3.032	3.019	2.710	3.2971
HCN-HCN	-2.414	-2.325	-2.250	-2.509	-2.390	-2.5542	0.846	0.950	0.861	1.060	0.837	1.1863
HF-HF	-4.088	-4.127	-4.755	-4.806	-4.860	-5.0719	1.885	2.073	2.457	2.564	2.241	2.7969
NH_3-NH_3	-1.968	-2.028	-2.423	-2.242	-2.254	-2.4157	1.191	1.297	1.638	1.470	1.364	1.6689
HF-CH_4	-2.137	-1.954	-2.046	-2.295	-2.213	-2.2998	0.738	0.710	0.750	0.916	0.764	0.9641
NH_3-CH_4	-0.634	-0.649	-0.725	-0.661	-0.682	-0.7019	0.233	0.271	0.341	0.279	0.251	0.3329
H_2O-CH_4	-0.487	-0.456	-0.497	-0.499	-0.506	-0.5058	0.194	0.191	0.231	0.231	0.202	0.2529
HCOH-HCOH	-5.604	-5.286	-5.478	-5.790	-6.116	-6.2680	3.813	3.961	4.153	4.389	4.161	5.0248
H_2O-C_2H_4	-3.562	-2.842	-2.904	-3.122	-3.522	-3.1875	2.377	1.894	1.885	2.080	2.351	2.1805
HCOH-C_2H_4	-1.737	-1.323	-1.316	-1.514	-1.669	-1.5274	1.346	1.053	1.041	1.211	1.293	1.2852
C_2H_2-C_2H_2(1)	-1.120	-0.864	-0.858	-0.992	-1.034	-0.9364	0.696	0.563	0.519	0.619	0.642	0.6201
NH_3-C_2H_4	-1.305	-1.011	-1.032	-1.140	-1.296	-1.1559	0.923	0.722	0.718	0.801	0.917	0.8477
C_2H_4-C_2H_4(1)	-1.014	-0.734	-0.739	-0.860	-0.999	-0.8766	0.902	0.663	0.641	0.751	0.889	0.7981
CH_4-C_2H_4	-0.294	-0.209	-0.217	-0.248	-0.289	-0.2523	0.214	0.159	0.161	0.180	0.211	0.1975
BH_3-CH_4	-1.841	-1.686	-1.751	-1.993	-1.971	-2.0338	1.068	0.969	1.043	1.243	1.143	1.3131
CH_4-C_2H_6(1)	-0.220	-0.193	-0.211	-0.274	-0.258	-0.2785	0.200	0.179	0.194	0.253	0.234	0.2674
CH_4-C_2H_6(2)	-0.155	-0.134	-0.141	-0.203	-0.189	-0.2067	0.144	0.128	0.133	0.193	0.176	0.2041
CH_4-CH_4	-0.146	-0.127	-0.137	-0.181	-0.171	-0.1858	0.137	0.121	0.131	0.172	0.161	0.1841
Ar-CH_4	-0.236	-0.212	-0.231	-0.287	-0.280	-0.3034	0.229	0.206	0.224	0.280	0.272	0.2979
Ar-C_2H_4	-0.971	-0.670	-0.651	-0.729	-0.930	-0.7707	0.950	0.652	0.630	0.712	0.910	0.7515
C_2H_4-C_2H_2	-2.357	-1.704	-1.626	-1.820	-2.187	-1.8775	2.197	1.592	1.503	1.706	2.038	1.7975
C_2H_4-C_2H_4(2)	-2.903	-2.019	-1.964	-2.181	-2.730	-2.2872	2.749	1.919	1.846	2.078	2.585	2.2228
C_2H_2-C_2H_2(2)	-1.822	-1.392	-1.297	-1.469	-1.664	-1.4812	1.654	1.268	1.168	1.344	1.510	1.3870

Table S17: Dispersion energy components [$E^{(2)}_{\mathrm{disp}}$, $E^{(2)}_{\mathrm{exch-disp}}(S^2)$, in milliHartree] for the **A24 data set**. MB stands for many-body SAPT2+(CCD). The basis set is aug-cc-pVDZ.

	$E^{(2)}_{\mathrm{disp}}$						$E^{(2)}_{\mathrm{exch-disp}}$					
	HF	GVB	CAS	PBE0	MB	CCSD	HF	GVB	CAS	PBE0	MB	CCSD
H_2O-NH_3	-4.884	-5.014	-5.375	-5.245	-5.450	-5.555	1.047	1.111	1.238	1.191	1.017	1.302
H_2O-H_2O	-3.471	-3.517	-3.810	-3.814	-3.975	-4.054	0.632	0.643	0.731	0.751	0.612	0.812
HCN-HCN	-2.566	-2.570	-2.589	-2.733	-2.772	-2.876	0.311	0.348	0.349	0.369	0.305	0.415
HF-HF	-2.390	-2.452	-2.648	-2.704	-2.832	-2.883	0.360	0.369	0.419	0.455	0.353	0.492
NH_3-NH_3	-3.144	-3.132	-3.390	-3.357	-3.432	-3.516	0.504	0.512	0.583	0.570	0.462	0.614
HF-CH_4	-2.013	-1.981	-2.072	-2.232	-2.272	-2.320	0.192	0.186	0.204	0.244	0.182	0.262
NH_3-CH_4	-1.486	-1.483	-1.644	-1.583	-1.605	-1.660	0.208	0.219	0.254	0.234	0.176	0.253
H_2O-CH_4	-1.197	-1.159	-1.279	-1.277	-1.310	-1.336	0.138	0.133	0.153	0.156	0.122	0.161
HCOH-HCOH	-6.255	-5.972	-6.267	-6.479	-6.846	-6.843	0.980	0.953	0.994	1.072	0.961	1.139
H_2O-C_2H_4	-3.472	-3.151	-3.287	-3.458	-3.610	-3.580	0.570	0.480	0.502	0.557	0.548	0.586
HCOH-C_2H_4	-3.305	-2.989	-3.187	-3.354	-3.434	-3.452	0.437	0.376	0.406	0.450	0.400	0.464
C_2H_2-C_2H_2(1)	-2.243	-2.031	-2.053	-2.204	-2.224	-2.224	0.275	0.246	0.247	0.271	0.260	0.281
NH_3-C_2H_4	-2.598	-2.335	-2.424	-2.551	-2.625	-2.613	0.374	0.309	0.321	0.358	0.346	0.369
C_2H_4-C_2H_4(1)	-3.508	-3.035	-3.161	-3.403	-3.434	-3.431	0.440	0.349	0.361	0.415	0.403	0.415
CH_4-C_2H_4	-1.530	-1.335	-1.393	-1.475	-1.502	-1.495	0.178	0.143	0.149	0.164	0.153	0.169
BH_3-CH_4	-4.061	-3.816	-3.955	-4.325	-4.211	-4.304	0.424	0.399	0.406	0.492	0.379	0.493
CH_4-C_2H_6(1)	-2.530	-2.393	-2.469	-2.692	-2.647	-2.709	0.207	0.186	0.198	0.246	0.178	0.245
CH_4-C_2H_6(2)	-1.870	-1.760	-1.808	-2.007	-1.964	-2.018	0.149	0.133	0.140	0.182	0.127	0.182
CH_4-CH_4	-1.637	-1.543	-1.591	-1.750	-1.713	-1.758	0.129	0.114	0.122	0.154	0.110	0.154
Ar-CH_4	-1.079	-1.049	-1.068	-1.136	-1.147	-1.166	0.083	0.079	0.085	0.095	0.074	0.099
Ar-C_2H_4	-1.098	-0.998	-1.002	-1.057	-1.100	-1.090	0.128	0.103	0.103	0.112	0.123	0.116
C_2H_4-C_2H_2	-4.100	-3.478	-3.479	-3.769	-3.864	-3.778	0.835	0.661	0.645	0.714	0.794	0.724
C_2H_4-C_2H_4(2)	-4.885	-4.046	-4.113	-4.439	-4.580	-4.473	1.035	0.795	0.794	0.870	0.985	0.895
C_2H_2-C_2H_2(2)	-3.422	-2.978	-2.934	-3.189	-3.244	-3.179	0.659	0.542	0.517	0.578	0.624	0.576

Table S18: The first-order energy contributions [$E^{(1)}_{\text{elst}}$ and $E^{(1)}_{\text{exch}}(S^2)$, in milliHartree] for the **TK21 database**. MB stands for many-body SAPT2+(CCD). The basis set is aug-cc-pVTZ.

	$E^{(1)}_{\text{elst}}$						$E^{(1)}_{\text{exch}}(S^2)$					
	HF	GVB	CAS	PBE0	MB	CCSD	HF	GVB	CAS	PBE0	MB	CCSD
F^--HF	-121.72	-120.1	-116.5	-116.2	-115.0	-114.9	105.1	123.4	122.7	122.0	127.0	126.2
F^--H_2O	-61.29	-61.56	-61.77	-60.93	-61.76	-61.31	52.49	57.00	58.83	60.98	63.39	62.62
Na^+-H_2O	-42.34	-41.87	-41.17	-40.12	-40.00	-39.44	13.14	13.89	14.78	14.48	14.28	14.27
HF-HF	-9.998	-9.730	-9.788	-9.574	-9.639	-9.437	7.260	8.045	8.376	8.828	8.864	8.902
CH_4-CH_4	-0.216	-0.196	-0.217	-0.301	-0.256	-0.242	0.843	0.766	0.818	1.223	0.933	0.974
H_2O-H_2O	-13.48	-13.46	-13.64	-13.10	-13.43	-13.12	11.51	12.64	13.19	13.15	13.34	13.43
NH_3-CH_4	-0.835	-0.768	-0.808	-0.986	-0.958	-0.922	1.518	1.535	1.666	1.976	1.692	1.742
NH_3-H_2O	-18.41	-18.75	-18.85	-17.79	-18.31	-17.91	17.01	18.96	19.25	18.51	18.71	18.83
N_2-N_2	-0.192	-0.272	-0.289	-0.234	-0.265	-0.251	0.475	0.486	0.518	0.525	0.484	0.510
C_2H_2-C_2H_2(PD)	-2.860	-2.283	-2.316	-2.495	-2.387	-2.347	2.844	2.598	2.574	2.783	2.797	2.745
C_2H_2-C_2H_2(S)	0.223	0.171	0.196	0.195	0.137	0.176	0.776	0.621	0.589	0.637	0.698	0.644
C_2H_2-C_2H_2(T)	-3.278	-2.641	-2.673	-2.830	-2.688	-2.677	3.308	3.061	2.920	3.137	3.148	3.097
C_2H_6-HCN	-1.075	-0.898	-0.916	-1.132	-1.162	-1.087	1.736	1.737	1.773	2.101	1.887	1.982
NCCN-NCCN	-3.423	-3.926	-3.494	-3.274	-3.630	-3.329	3.574	3.638	3.555	4.014	3.776	3.840
P_2-P_2	-1.308	-1.041	-0.970	-0.961	-1.040	-0.980	3.468	2.906	2.822	2.608	3.154	2.907
N_2O-He(GM)	-0.073	-0.076	-0.071	-0.076	-0.082	-0.082	0.343	0.349	0.344	0.374	0.367	0.384
N_2O-He(LM)	-0.028	-0.034	-0.028	-0.035	-0.034	-0.034	0.147	0.166	0.148	0.181	0.178	0.180
CO_2-He(GM)	-0.060	-0.065	-0.070	-0.070	-0.076	-0.075	0.322	0.333	0.373	0.382	0.373	0.386
CO_2-He(LM)	-0.020	-0.024	-0.020	-0.026	-0.026	-0.025	0.110	0.131	0.111	0.142	0.133	0.138
Ar -Ar	-0.077	-0.081	-0.084	-0.081	-0.089	-0.086	0.260	0.272	0.278	0.269	0.285	0.286
PCCP-PCCP	-3.448	-2.885	-3.073	-2.839	-3.112	-2.881	8.208	7.460	8.258	6.927	7.729	7.449

Table S19: Induction energy components [$E^{(2)}_{\rm ind}$, $E^{(2)}_{\rm exch-ind}(S^2)$, in milliHartree] for the **TK21 data set**. Systems from the TK21/S_2 subset are marked in gray. MB stands for many-body SAPT2+(CCD). The basis set is aug-cc-pVTZ.

	$E^{(2)}_{\rm ind}$						$E^{(2)}_{\rm exch-ind}$					
	HF	GVB	CAS	PBE0	MB	CCSD	HF	GVB	CAS	PBE0	MB	CCSD
F^--HF	-92.62	-100.6	-106.0	-109.96	-109.81	-112.43	35.96	43.08	47.11	52.84	42.63	50.31
F^--H_2O	-37.284	-37.877	-43.104	-46.29	-45.14	-47.06	18.65	19.74	23.82	27.95	22.58	27.09
Na^+-H_2O	-23.428	-23.448	-26.341	-26.31	-25.98	-26.51	12.93	13.20	15.34	15.15	14.34	14.85
HF-HF	-3.908	-3.933	-4.475	-4.633	-4.548	-4.669	1.833	1.997	2.311	2.497	2.133	2.554
CH_4-CH_4	-0.098	-0.084	-0.093	-0.176	-0.111	-0.120	0.092	0.080	0.088	0.172	0.104	0.119
H_2O-H_2O	-4.974	-5.081	-5.897	-5.851	-5.734	-5.929	2.791	3.014	3.603	3.646	3.217	3.737
NH_3-CH_4	-0.374	-0.360	-0.405	-0.506	-0.404	-0.418	0.221	0.217	0.261	0.355	0.239	0.282
NH_3-H_2O	-8.223	-8.650	-9.873	-9.396	-8.994	-9.243	4.783	5.332	6.239	6.051	5.231	5.821
N_2-N_2	-0.072	-0.079	-0.095	-0.089	-0.081	-0.090	0.066	0.068	0.083	0.081	0.073	0.083
C_2H_2-C_2H_2(PD)	-0.909	-0.707	-0.692	-0.822	-0.830	-0.759	0.692	0.556	0.526	0.634	0.631	0.610
C_2H_2-C_2H_2(S)	-0.259	-0.186	-0.174	-0.197	-0.216	-0.188	0.190	0.137	0.124	0.142	0.158	0.141
C_2H_2-C_2H_2(T)	-1.096	-0.851	-0.843	-0.965	-0.984	-0.895	0.685	0.556	0.513	0.600	0.615	0.580
C_2H_6-HCN	-0.901	-0.749	-0.770	-0.947	-0.874	-0.860	0.269	0.246	0.252	0.368	0.261	0.325
NCCN-NCCN	-1.191	-1.277	-1.189	-1.383	-1.250	-1.356	0.787	0.843	0.798	1.027	0.825	1.014
P_2-P_2	-1.908	-1.422	-1.291	-1.331	-1.541	-1.328	1.823	1.359	1.244	1.274	1.472	1.310
N_2O-He(GM)	-0.049	-0.059	-0.046	-0.047	-0.045	-0.049	0.028	0.031	0.030	0.030	0.026	0.033
N_2O-He(LM)	-0.014	-0.020	-0.011	-0.012	-0.010	-0.012	0.007	0.009	0.008	0.009	0.005	0.010
CO_2-He(GM)	-0.051	-0.055	-0.046	-0.048	-0.045	-0.047	0.024	0.026	0.027	0.027	0.021	0.030
CO_2-He(LM)	-0.010	-0.011	-0.009	-0.010	-0.009	-0.010	0.005	0.006	0.005	0.006	0.004	0.006
Ar -Ar	-0.086	-0.090	-0.097	-0.091	-0.098	-0.100	0.085	0.088	0.095	0.089	0.096	0.099
PCCP-PCCP	-4.406	-3.647	-4.466	-3.534	-3.884	-3.524	4.073	3.453	4.195	3.298	3.590	3.351

Table S20: Dispersion energy components [$E^{(2)}_{\mathrm{disp}}$, $E^{(2)}_{\mathrm{exch-disp}}(S^2)$, in milliHartree] for the **TK21 data set**. Systems from the TK21/S_2 subset are marked in gray. MB stands for many-body SAPT2+(CCD). The basis set is aug-cc-pVTZ.

	$E^{(2)}_{\mathrm{disp}}$						$E^{(2)}_{\mathrm{exch-disp}}$					
	HF	GVB	CAS	PBE0	MB	CCSD	HF	GVB	CAS	PBE0	MB	CCSD
F^--HF	-20.67	-22.61	-23.35	-23.72	-25.09	-25.38	6.272	5.914	6.437	7.912	6.313	8.377
F^--H_2O	-12.81	-13.09	-14.25	-14.83	-15.21	-15.58	4.065	4.013	4.458	5.154	3.942	5.367
Na^+-H_2O	-0.779	-0.777	-0.860	-0.870	-0.848	-0.876	0.145	0.147	0.165	0.169	0.135	0.164
HF-HF	-2.673	-2.749	-2.959	-3.078	-3.111	-3.179	0.400	0.414	0.465	0.513	0.380	0.527
CH_4-CH_4	-1.560	-1.472	-1.525	-1.744	-1.617	-1.666	0.114	0.100	0.109	0.161	0.097	0.133
H_2O-H_2O	-4.273	-4.345	-4.701	-4.741	-4.796	-4.902	0.808	0.830	0.938	0.967	0.767	1.003
NH_3-CH_4	-1.819	-1.765	-1.882	-2.018	-1.920	-1.978	0.184	0.176	0.200	0.238	0.159	0.212
NH_3-H_2O	-5.822	-5.990	-6.403	-6.344	-6.358	-6.481	1.264	1.346	1.492	1.476	1.204	1.505
N_2-N_2	-0.721	-0.702	-0.727	-0.747	-0.736	-0.753	0.045	0.044	0.050	0.050	0.041	0.049
C_2H_2-C_2H_2(PD)	-2.480	-2.251	-2.278	-2.467	-2.438		0.306	0.271	0.273	0.309	0.290	
C_2H_2-C_2H_2(S)	-1.280	-1.105	-1.084	-1.190	-1.183		0.139	0.106	0.099	0.117	0.123	
C_2H_2-C_2H_2(T)	-2.417	-2.201	-2.219	-2.388	-2.361		0.305	0.276	0.276	0.302	0.283	
C_2H_6-HCN	-1.812	-1.735	-1.809	-1.973	-1.901		0.138	0.137	0.149	0.177	0.125	
NCCN-NCCN	-3.310	-3.175	-3.202	-3.431	-3.436		0.361	0.360	0.361	0.417	0.355	
P_2-P_2	-3.768	-3.333	-3.267	-3.386	-3.676	-3.516	0.552	0.455	0.419	0.443	0.548	0.440
N_2O-He(GM)	-0.510	-0.505	-0.509	-0.541	-0.541	-0.552	0.022	0.023	0.022	0.025	0.019	0.025
N_2O-He(LM)	-0.262	-0.268	-0.262	-0.282	-0.288	-0.290	0.010	0.010	0.009	0.011	0.008	0.011
CO_2-He(GM)	-0.438	-0.441	-0.457	-0.481	-0.483	-0.494	0.018	0.020	0.021	0.022	0.016	0.023
CO_2-He(LM)	-0.203	-0.207	-0.204	-0.223	-0.223	-0.227	0.007	0.007	0.007	0.008	0.005	0.008
Ar -Ar	-0.515	-0.514	-0.519	-0.538	-0.550	-0.554	0.025	0.025	0.026	0.027	0.023	0.028
PCCP-PCCP	-9.177	-8.168	-8.852	-8.506	-9.240		1.416	1.220	1.439	1.240	1.507	

Table S21: The first-order energy contributions [$E^{(1)}_{\mathrm{elst}}$ and $E^{(1)}_{\mathrm{exch}}(S^2)$, in milliHartree] for the **A24 database**. MB stands for many-body SAPT2+(CCD). The basis set is aug-cc-pVTZ.

	$E^{(1)}_{\mathrm{elst}}$						$E^{(1)}_{\mathrm{exch}}(S^2)$					
	HF	GVB	CAS	PBE0	MB	CCSD	HF	GVB	CAS	PBE0	MB	CCSD
H_2O-NH_3	-18.00	-18.33	-18.44	-17.29	-17.91	-17.51	16.33	18.20	18.48	17.55	17.94	18.07
H_2O-H_2O	-12.94	-12.89	-13.02	-12.49	-12.78	-12.48	10.53	11.56	12.03	12.01	12.17	12.24
HCN-HCN	-10.128	-9.88	-9.51	-9.29	-9.539	-9.173	6.030	6.833	6.375	6.816	6.566	6.907
HF-HF	-10.59	-10.28	-10.36	-10.13	-10.20	-9.983	7.965	8.841	9.173	9.630	9.712	9.751
NH_3-NH_3	-7.592	-7.633	-7.822	-7.496	-7.810	-7.573	6.518	6.931	7.433	7.167	7.189	7.301
HF-CH_4	-1.946	-1.591	-1.551	-2.384	-2.304	-2.271	3.473	3.526	3.614	4.089	4.045	4.128
NH_3-CH_4	-1.455	-1.412	-1.457	-1.521	-1.585	-1.527	2.086	2.398	2.565	2.277	2.294	2.352
H_2O-CH_4	-1.092	-0.991	-1.026	-1.134	-1.152	-1.111	1.532	1.627	1.772	1.714	1.707	1.716
HCOH-HCOH	-12.47	-10.60	-10.12	-10.41	-10.37	-10.00	12.17	12.72	12.32	13.15	13.52	13.52
H_2O-C_2H_4	-6.354	-5.175	-5.336	-5.424	-5.623	-5.441	7.017	6.331	6.295	6.509	7.040	6.663
HCOH-C_2H_4	-3.299	-2.565	-2.526	-2.748	-2.788	-2.666	4.050	3.715	3.674	4.013	4.109	4.016
C_2H_2-C_2H_2(1)	-3.408	-2.738	-2.779	-2.946	-2.791	-2.780	3.494	3.228	3.085	3.349	3.322	3.269
NH_3-C_2H_4	-3.089	-2.496	-2.557	-2.672	-2.753	-2.634	3.842	3.406	3.414	3.537	3.786	3.572
C_2H_4-C_2H_4(1)	-1.526	-1.257	-1.284	-1.395	-1.447	-1.351	3.447	3.040	2.980	3.242	3.397	3.251
CH_4-C_2H_4	-0.762	-0.572	-0.582	-0.676	-0.703	-0.659	1.544	1.323	1.343	1.363	1.490	1.392
BH_3-CH_4	-2.267	-2.008	-2.025	-2.494	-2.462	-2.422	5.833	5.497	5.692	6.518	6.236	6.407
CH_4-C_2H_6(1)	-0.525	-0.475	-0.511	-0.591	-0.609	-0.582	1.801	1.690	1.783	2.087	1.968	2.033
CH_4-C_2H_6(2)	-0.334	-0.303	-0.323	-0.387	-0.408	-0.383	1.261	1.158	1.208	1.500	1.403	1.460
CH_4-CH_4	-0.294	-0.266	-0.292	-0.328	-0.347	-0.329	1.122	1.024	1.089	1.307	1.237	1.290
Ar-CH_4	-0.256	-0.243	-0.256	-0.286	-0.302	-0.290	0.864	0.838	0.870	0.956	0.947	0.975
Ar-C_2H_4	-0.470	-0.366	-0.358	-0.384	-0.424	-0.382	1.235	1.011	0.991	1.042	1.162	1.066
C_2H_4-C_2H_2	-0.571	-0.559	-0.465	-0.529	-0.744	-0.537	6.358	5.194	5.046	5.412	5.893	5.458
C_2H_4-C_2H_4(2)	-1.019	-0.894	-0.786	-0.830	-1.090	-0.850	7.597	6.030	5.911	6.306	7.127	6.469
C_2H_2-C_2H_2(2)	0.535	0.317	0.408	0.360	0.094	0.304	5.336	4.501	4.332	4.683	4.880	4.628

Table S22: Induction energy components [$E^{(2)}_{\text{ind}}$, $E^{(2)}_{\text{exch-ind}}(S^2)$, in milliHartree] for the **A24 data set**. MB stands for many-body SAPT2+(CCD). The basis set is aug-cc-pVTZ.

	$E^{(2)}_{\text{ind}}$						$E^{(2)}_{\text{exch-ind}}$					
	HF	GVB	CAS	PBE0	MB	CCSD	HF	GVB	CAS	PBE0	MB	CCSD
H_2O-NH_3	-7.926	-8.341	-9.524	-8.441	-8.666	-8.906	4.603	5.138	6.015	5.245	5.032	5.601
H_2O-H_2O	-4.542	-4.628	-5.339	-5.300	-5.204	-5.359	2.477	2.677	3.187	3.223	2.838	3.301
HCN-HCN	-2.480	-2.374	-2.318	-2.599	-2.436	-2.562	0.888	0.988	0.908	1.124	0.872	1.172
HF-HF	-4.288	-4.316	-4.904	-5.056	-4.991	-5.121	2.034	2.222	2.558	2.744	2.367	2.831
NH_3-NH_3	-2.058	-2.105	-2.507	-2.361	-2.286	-2.390	1.258	1.355	1.697	1.559	1.398	1.634
HF-CH_4	-2.229	-2.030	-2.147	-2.413	-2.291	-2.365	0.764	0.731	0.774	0.951	0.786	0.958
NH_3-CH_4	-0.651	-0.675	-0.745	-0.684	-0.685	-0.691	0.252	0.286	0.360	0.301	0.265	0.325
H_2O-CH_4	-0.513	-0.487	-0.528	-0.529	-0.523	-0.517	0.219	0.214	0.257	0.257	0.223	0.263
HCOH-HCOH	-5.851	-5.525	-5.713	-6.127	-6.196	-6.215	4.048	4.208	4.376	4.712	4.287	5.000
H_2O-C_2H_4	-3.690	-2.951	-3.012	-3.122	-3.515	-3.176	2.478	1.979	1.963	2.080	2.361	2.151
HCOH-C_2H_4	-1.775	-1.351	-1.347	-1.569	-1.636	-1.489	1.382	1.082	1.070	1.262	1.274	1.239
C_2H_2-C_2H_2(1)	-1.162	-0.899	-0.893	-1.036	-1.042	-0.947	0.726	0.588	0.544	0.651	0.651	0.614
NH_3-C_2H_4	-1.361	-1.059	-1.080	-1.197	-1.291	-1.150	0.975	0.765	0.760	0.849	0.925	0.838
C_2H_4-C_2H_4	-1.054	-0.766	-0.769	-0.900	-0.975	-0.854	0.942	0.695	0.671	0.789	0.872	0.776
CH_4-C_2H_4	-0.310	-0.224	-0.232	-0.263	-0.289	-0.254	0.233	0.175	0.177	0.196	0.217	0.199
BH_3-CH_4	-2.034	-1.861	-1.943	-2.209	-2.099	-2.142	1.225	1.110	1.189	1.424	1.265	1.433
CH_4-C_2H_6(1)	-0.216	-0.188	-0.206	-0.269	-0.241	-0.256	0.196	0.174	0.189	0.248	0.219	0.247
CH_4-C_2H_6(2)	-0.157	-0.135	-0.143	-0.203	-0.182	-0.195	0.147	0.129	0.135	0.194	0.170	0.195
CH_4-CH_4	-0.136	-0.116	-0.128	-0.167	-0.153	-0.164	0.128	0.111	0.122	0.159	0.143	0.164
Ar-CH_4	-0.226	-0.202	-0.220	-0.273	-0.257	-0.274	0.219	0.196	0.214	0.267	0.250	0.272
Ar-C_2H_4	-0.985	-0.679	-0.656	-0.747	-0.880	-0.727	0.964	0.662	0.635	0.727	0.861	0.711
C_2H_4-C_2H_2	-2.462	-1.776	-1.690	-1.942	-2.132	-1.827	2.308	1.671	1.573	1.831	1.998	1.755
C_2H_4-C_2H_4(2)	-3.033	-2.107	-2.043	-2.320	-2.657	-2.221	2.895	2.022	1.938	2.226	2.536	2.171
C_2H_2-C_2H_2(2)	-1.918	-1.459	-1.359	-1.582	-1.636	-1.453	1.749	1.337	1.231	1.455	1.492	1.363

Table S23: Dispersion energy components [$E^{(2)}_{\rm disp}$, $E^{(2)}_{\rm exch-disp}(S^2)$, in milliHartree] for the **A24 data set**. MB stands for many-body SAPT2+(CCD). The basis set is aug-cc-pVTZ.

	$E^{(2)}_{\rm disp}$					$E^{(2)}_{\rm exch-disp}$			
	HF	GVB	CAS	PBE0	MB	HF	GVB	CAS	PBE0
H_2O-NH_3	-5.667	-5.828	-6.229	-5.245	-6.185	1.218	1.298	1.438	1.191
H_2O-H_2O	-4.036	-4.101	-4.428	-4.469	-4.521	0.742	0.762	0.859	0.886
HCN-HCN	-2.882	-2.890	-2.918	-3.088	-3.054	0.377	0.419	0.422	0.447
HF-HF	-2.841	-2.926	-3.142	-3.263	-3.308	0.436	0.452	0.507	0.556
NH_3-NH_3	-3.617	-3.608	-3.899	-3.874	-3.864	0.595	0.606	0.687	0.675
HF-CH_4	-2.383	-2.355	-2.461	-2.679	-2.661	0.245	0.240	0.261	0.313
NH_3-CH_4	-1.643	-1.673	-1.821	-1.758	-1.729	0.239	0.253	0.292	0.270
H_2O-CH_4	-1.342	-1.329	-1.441	-1.443	-1.436	0.163	0.158	0.182	0.185
HCOH-HCOH	-7.250	-6.952	-7.266	-7.553	-7.799	1.163	1.140	1.182	1.276
H_2O-C_2H_4	-3.931	-3.586	-3.734	-3.458	-4.020	0.655	0.561	0.584	0.557
HCOH-C_2H_4	-3.700	-3.357	-3.570	-3.777	-3.782	0.499	0.434	0.466	0.518
C_2H_2-C_2H_2(1)	-2.501	-2.273	-2.295	-2.476	-2.440	0.321	0.290	0.290	0.320
NH_3-C_2H_4	-2.879	-2.602	-2.699	-2.849	-2.858	0.425	0.357	0.370	0.411
C_2H_4-C_2H_4	-3.846	-3.341	-3.471	-3.745	-3.716	0.497	0.399	0.411	0.472
CH_4-C_2H_4	-1.647	-1.467	-1.506	-1.594	-1.587	0.198	0.161	0.167	0.184
BH_3-CH_4	-4.698	-4.434	-4.636	-5.011	-4.862	0.529	0.497	0.504	0.611
CH_4-C_2H_6(1)	-2.783	-2.641	-2.727	-2.969	-2.883	0.237	0.213	0.229	0.282
CH_4-C_2H_6(2)	-2.054	-1.937	-1.995	-2.210	-2.134	0.170	0.151	0.160	0.208
CH_4-CH_4	-1.812	-1.710	-1.771	-1.943	-1.878	0.147	0.130	0.140	0.176
Ar-CH_4	-1.259	-1.222	-1.252	-1.334	-1.324	0.098	0.093	0.085	0.112
Ar-C_2H_4	-1.310	-1.182	-1.195	-1.272	-1.296	0.152	0.122	0.120	0.135
C_2H_4-C_2H_2	-4.591	-3.902	-3.893	-4.249	-4.258	0.919	0.731	0.712	0.796
C_2H_4-C_2H_4(2)	-5.350	-4.443	-4.507	-4.892	-4.949	1.118	0.865	0.861	0.949
C_2H_2-C_2H_2(2)	-3.911	-3.408	-3.348	-3.671	-3.642	0.741	0.610	0.583	0.659

Table S24: Summary of error statistics (in percent) for the SAPT interaction energy for dimers of the **A24** and **TK21** datasets. Errors of the SAPT interaction energies corrected for the δ_{HF} term ($E_{\mathrm{int}}^{\mathrm{SAPT}+\delta_{\mathrm{HF}}}$) are given with respect to the supermolecular CCSD(T) results calculated in the same basis set. The 2+(CCD) notation refers to the SAPT2+(CCD) scheme. All exchange energy components are included in the S^2 approximation. The basis set is aug-cc-pVTZ.

	A24				
$^{\ddagger}E_{\mathrm{int}}^{\mathrm{SAPT}+\delta_{\mathrm{HF}}}$	HF	GVB	CAS	PBE0	2+(CCD)
$\overline{\Delta}$	-8.47	2.07	-1.09	-3.36	-5.20
σ	20.48	9.26	9.33	6.59	10.38
$\overline{\Delta}_{\mathrm{abs}}$	17.90	6.72	7.79	5.86	10.22
Δ_{max}	50.01	25.05	19.53	13.23	23.96
	* TK21				
$^{\ddagger}E_{\mathrm{int}}^{\mathrm{SAPT}+\delta_{\mathrm{HF}}}$	HF	GVB	CAS	PBE0	2+(CCD)
$\overline{\Delta}$	-10.43	-1.36	-0.61	-1.23	-8.64
σ	10.70	10.13	9.69	9.76	7.62
$\overline{\Delta}_{\mathrm{abs}}$	11.90	8.12	7.82	5.87	9.74
Δ_{max}	29.26	13.97	26.16	31.85	24.37

‡ errors with respect to supermolecular CCSD (T)
* Table 3 in the manuscript is for the S_2 subset of TK21

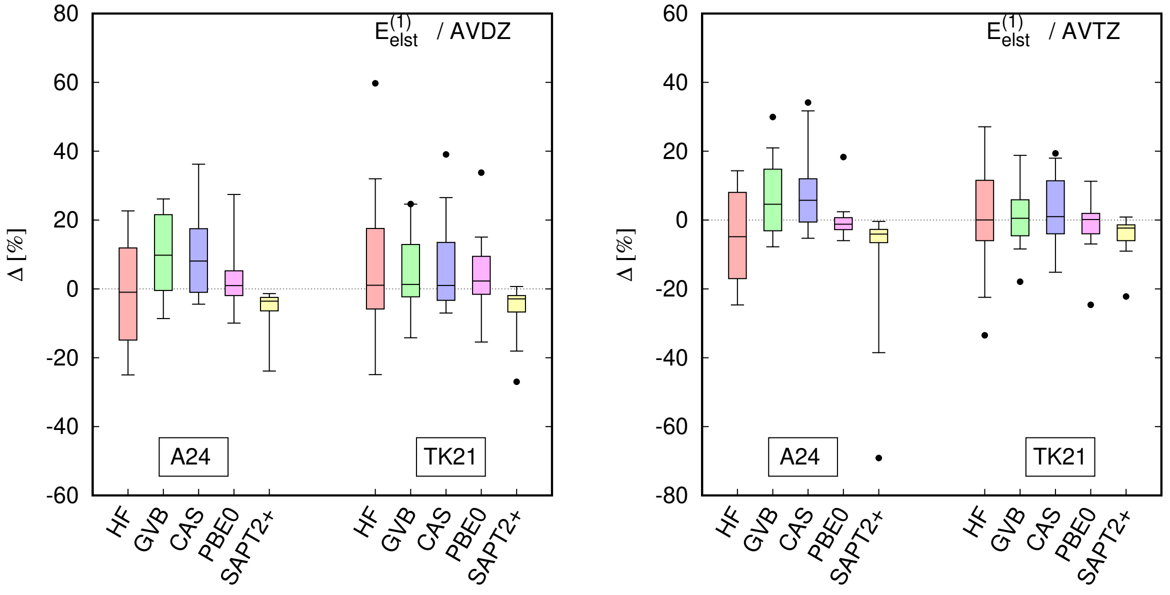

Figure S5: Box plots of relative percent errors in the calculated **electrostatic** energies for dimers of the A24 and TK21 data set. HF, GVB and CAS denote wavefunction description of the monomers. Errors are given with respect to the SAPT(CCSD) reference. The box and outer fences encompass 50% and 95% of the distribution, respectively.

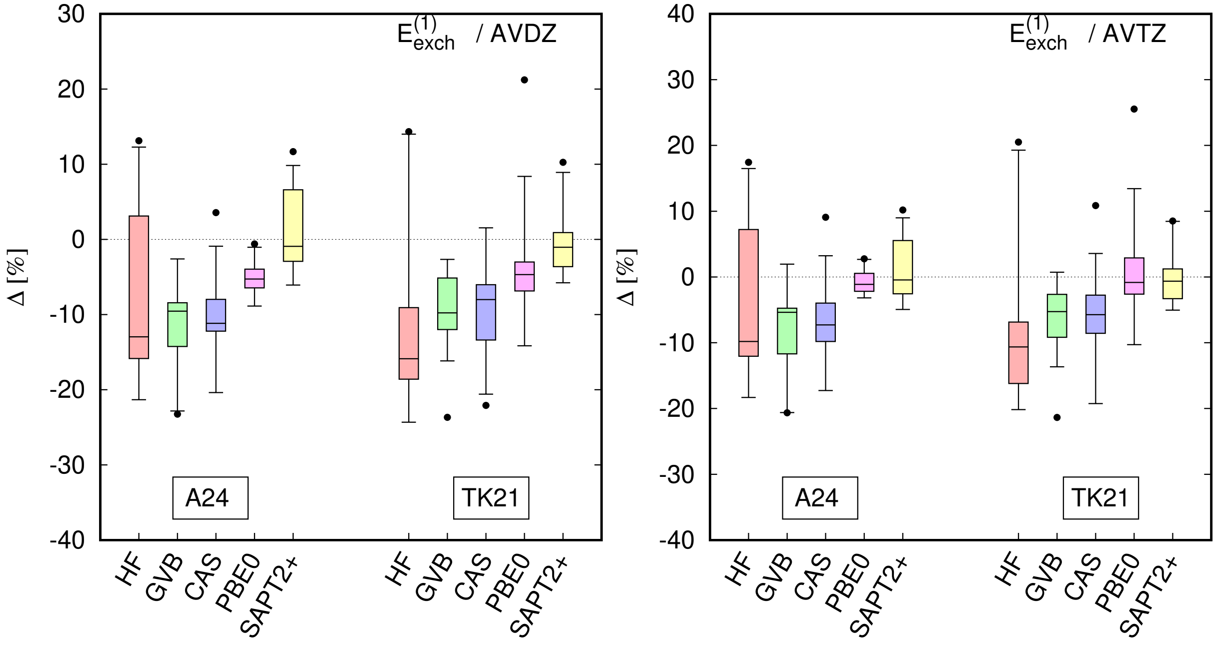

Figure S6: Box plots of relative percent errors in the calculated **exchange** energies for dimers of the A24 and TK21 data set. HF, GVB and CAS denote wavefunction description of the monomers. Errors are given with respect to the SAPT(CCSD) reference. The box and outer fences encompass 50% and 95% of the distribution, respectively.

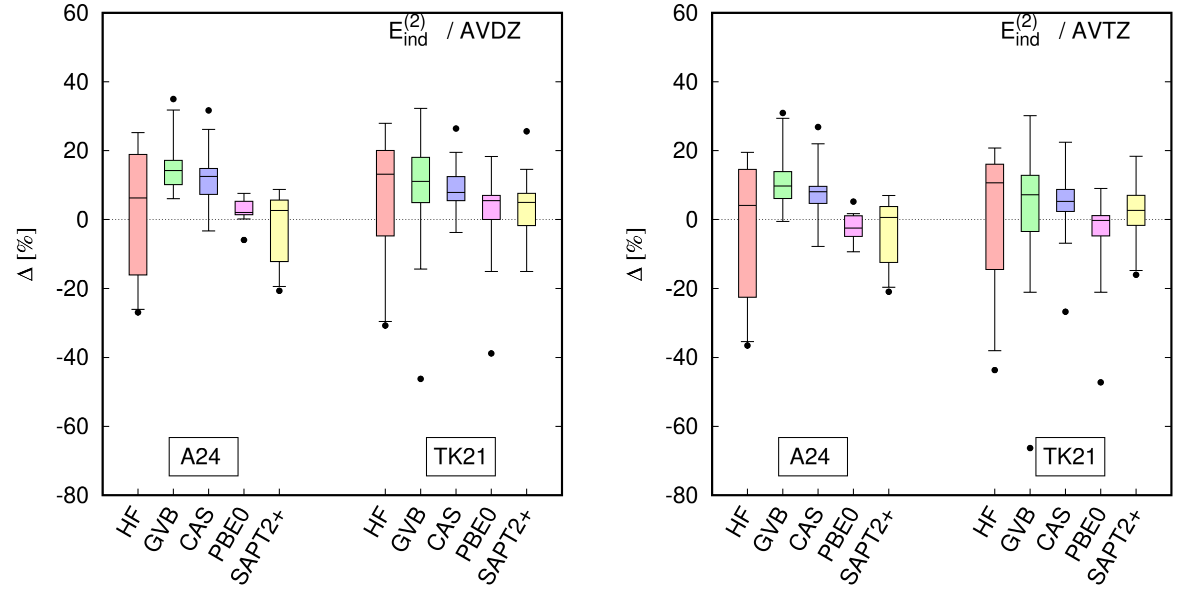

Figure S7: Box plots of relative percent errors in the calculated **induction** energies for dimers of the A24 and TK21 data set. HF, GVB and CAS denote wavefunction description of the monomers. Errors are given with respect to the SAPT(CCSD) reference. The box and outer fences encompass 50% and 95% of the distribution, respectively.

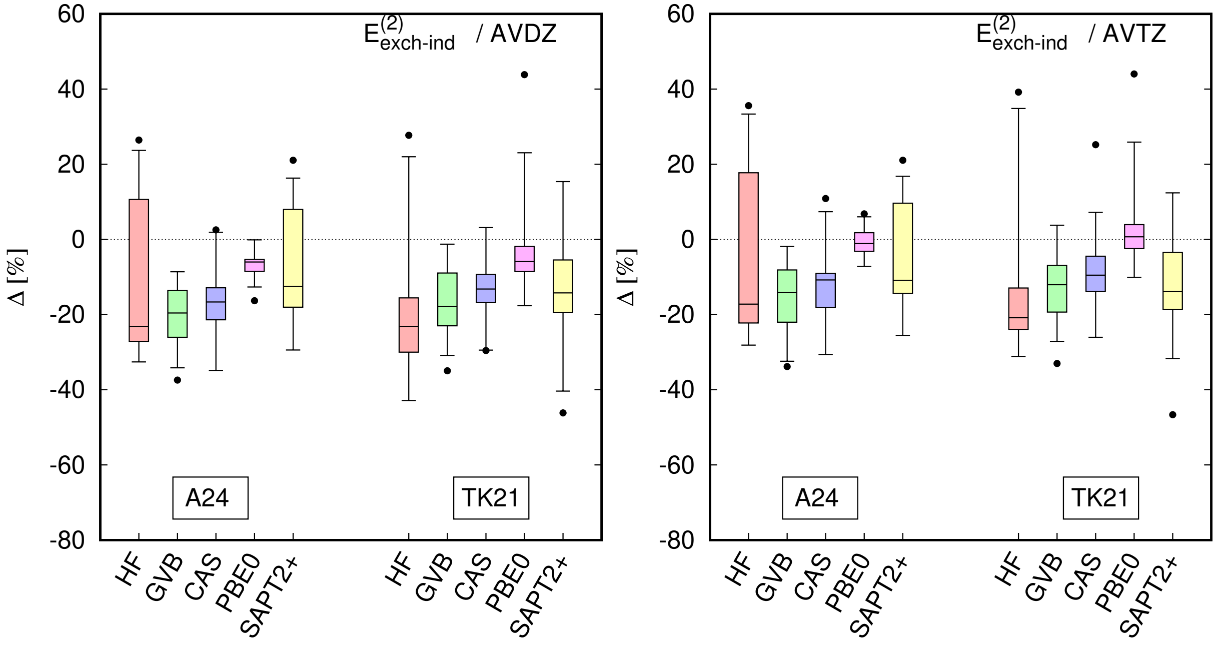

Figure S8: Box plots of relative percent errors in the calculated **exchange-induction** energies for dimers of the A24 and TK21 data set. HF, GVB and CAS denote wavefunction description of the monomers. Errors are given with respect to the SAPT(CCSD) reference. The box and outer fences encompass 50% and 95% of the distribution, respectively.

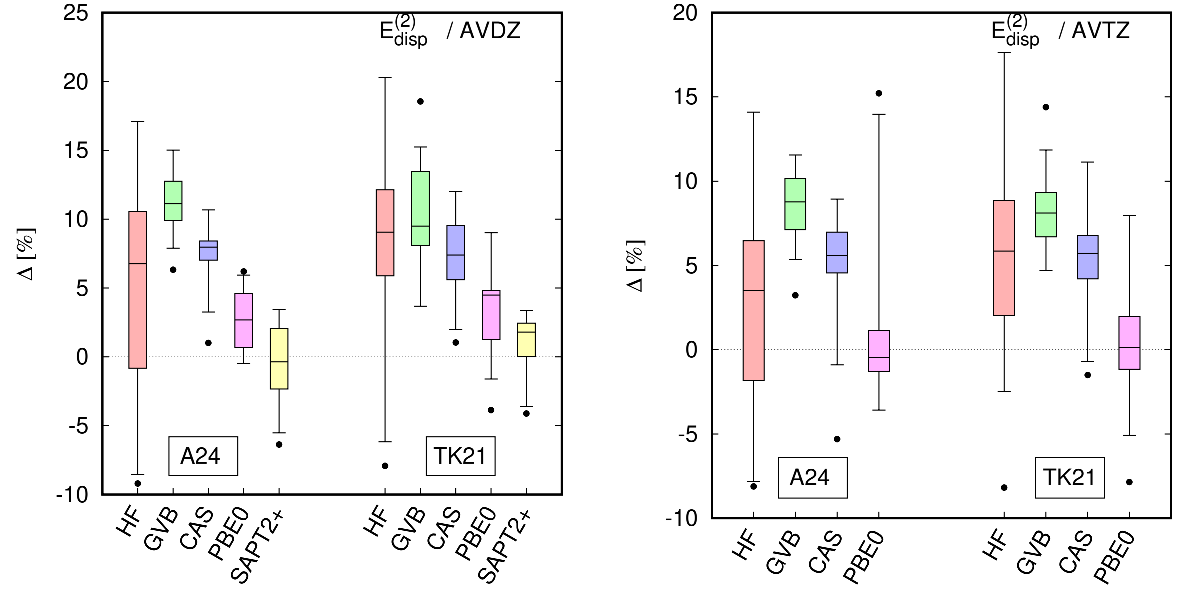

Figure S9: Box plots of relative percent errors in the calculated **dispersion** energies for dimers of the A24 and TK21 data set. HF, GVB and CAS denote wavefunction description of the monomers. Errors are given with respect to the SAPT(CCSD) reference. The box and outer fences encompass 50% and 95% of the distribution, respectively.

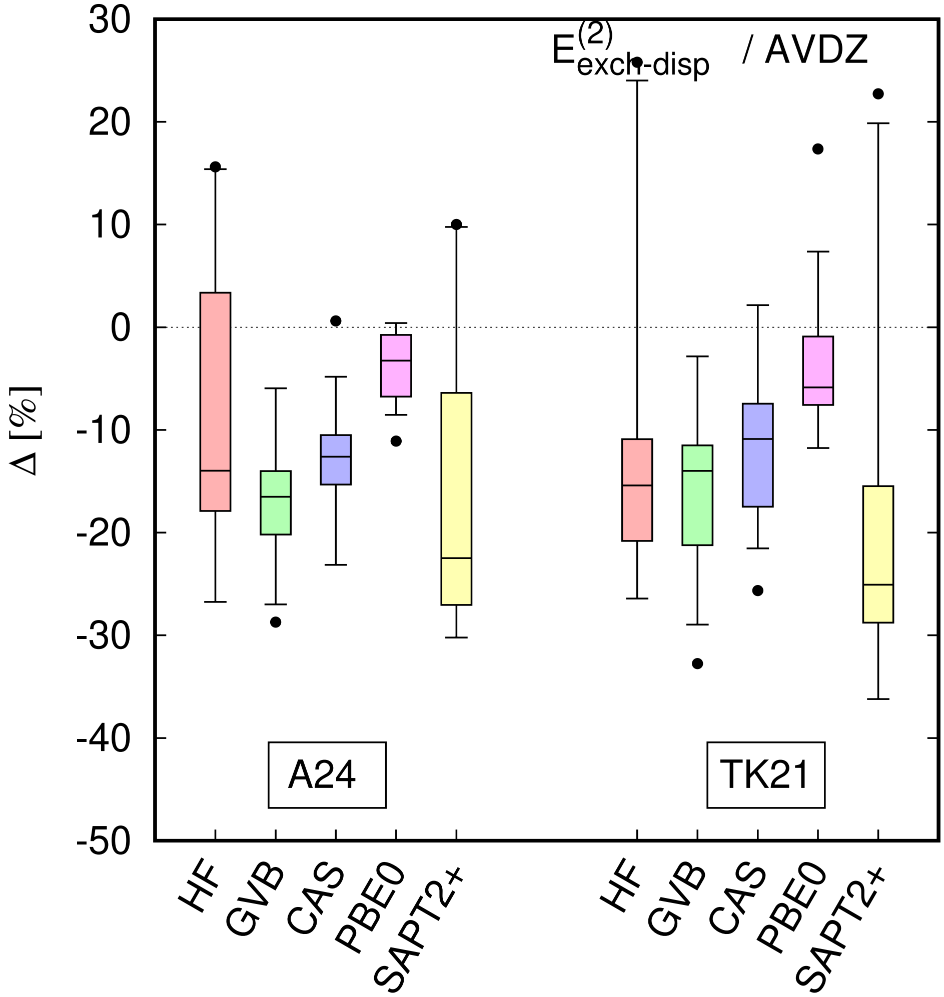

Figure S10: Box plots of relative percent errors in the calculated **exchange-dispersion** energies for dimers of the A24 and TK21 data set. HF, GVB and CAS denote wavefunction description of the monomers. Errors are given with respect to the SAPT(CCSD) reference. The box and outer fences encompass 50% and 95% of the distribution, respectively.

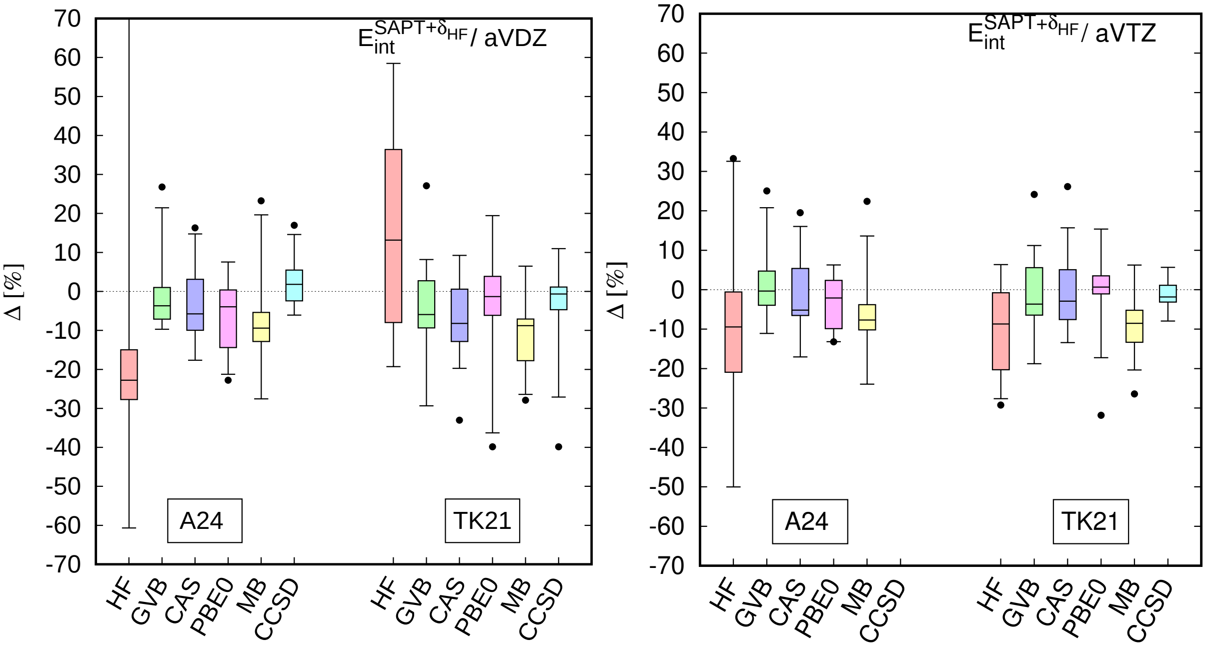

Figure S11: Box plots of relative percent errors in the calculated SAPT interaction energies (including the δ_{HF} term) for dimers of the A24 and TK21 data set. MB stands for SAPT2+(CCD). Errors are given with respect to the supermolecular CCSD(T) reference (in the same basis set as SAPT results). The SAPT(CCSD)/AVTZ results are given for the S_2 of the TK21 data set. The box and outer fences encompass 50% and 95% of the distribution, respectively.
